# Thiazolopyrimidine derivatives as novel class of small molecule tyrosinase inhibitor

**DOI:** 10.1186/s13065-023-01077-z

**Published:** 2023-11-19

**Authors:** Nastaran Ghasemi, Shahram Moradi, Aida Iraji, Mohammad Mahdavi

**Affiliations:** 1 Faculty of Chemistry, Tehran North Branch, Islamic Azad University, Tehran, Iran; 2 Stem Cells Technology Research Center, Shiraz University of Medical Sciences, Shiraz, Iran; 3 Research Center for Traditional Medicine and History of Medicine, Department of Persian Medicine, School of Medicine, Shiraz University of Medical Sciences, Shiraz, Iran; 4 Endocrinology and Metabolism Research Center, Endocrinology and Metabolism Clinical Sciences Institute, Tehran University of Medical Sciences, Tehran, Iran

**Keywords:** Thiazolopyrimidine, Tyrosinase, Synthesis, Molecular dynamic simulation

## Abstract

Tyrosinase, the rate-limiting enzyme of melanogenesis, plays a crucial role in hyperpigmentation. As a result, in this study, a novel class of thiazolopyrimidine derivatives was developed and synthesized as tyrosinase inhibitor. The structure of derivatives was characterized using various spectroscopy techniques, including FTIR, Mass, ^1^H-NMR, and ^13^C-NMR. Next, the inhibitory activities of all derivatives were examined against tyrosinase, and, **6a** as the most potent compound, exhibited an IC_50_ value of 28.50 µM. Furthermore, the kinetic study of **6a** was performed to better understand the inhibitory mechanism and its type of inhibition. The UV/Vis spectra analysis was also executed to provide valuable evidence supporting the inhibitory mechanism of compound **6a** in the context of tyrosinase inhibition. Also, molecular docking and dynamic molecular study of **6a** were executed to study its interactions within the enzyme’s binding site.

**Supplementary Information:**

The online version contains supplementary material available at 10.1186/s13065-023-01077-z.

## Introduction

Melanin is a complex biological polymer that provides color to the skin, hair, and eyes. The primary function of melanin is to protect the skin from the harmful effects of the sun's ultraviolet (UV) radiation. Although its structure is not fully understood, it was proposed to comprise various forms of melanin molecules, including eumelanin and/or pheomelanin, plus other organic compounds [1]. Melanocytes, specialized cells found in the skin, hair follicles, and various other tissues, are responsible for producing melanin. However, when melanin production becomes excessive, known as hyperpigmentation, it results in dark spots or patches on the skin. Several disorders associated with hyperpigmentation include melasma, Riehl's melanosis, post-inflammatory hyperpigmentation (PIH), solar lentigo, ashy dermatosis, and pigmented tumors [2, 3].

Furthermore, there is a correlation between several neurodegenerative diseases, including Alzheimer's, Parkinson's, and Huntington's disease [4]. Hyperpigmentation disorders significantly impact a person's appearance and may cause distress or self-consciousness. Understanding melanin production and regulation mechanisms is crucial for developing effective treatments and interventions to manage hyperpigmentation disorders [5].

Melanin production is a complex process involving several enzymes and chemical reactions, and amongst tyrosinase plays a key role in melanin production. Tyrosinase is a copper-containing enzyme that catalyzes the conversion of the amino acid tyrosine into 3,4-dihydroxyphenylalanine (Dopa) and the subsequent oxidation of Dopa to dopaquinone, a precursor to both eumelanin and pheomelanin two known types of melanin. Tyrosinase is also involved in the later stages of melanin production, which converts dopaquinone into melanin [6, 7]. Furthermore, tyrosinase is known to be involved in enzymatic browning processes observed in fruits and vegetables. This enzymatic browning can lead to faster spoilage of the product, resulting in changes in texture and flavor. This phenomenon imposes an economic burden on society due to food wastage [8].

The active site of tyrosinase consists of two copper ions and a binuclear copper-binding site are coordinated by several amino acid residues, including histidine, aspartic acid, and cysteine, which are essential for the stability and function of the enzyme. The active site of human tyrosinase is highly conserved among different species [5]. Over the past few years, numerous tyrosinase inhibitors have been developed, such as Hydroquinone, Arbutin, Kojic acid, and Vitamin C, commonly used in cosmetics, dermatology, and the agricultural industry. However, these inhibitors are associated with potential side effects, including skin irritation, ochronosis and allergic reactions [9, 10]. As a result, efforts have been made to develop novel tyrosinase inhibitors for their potential applications to overcome the challenges of low efficacy and side effect.

**Fig. 1:**
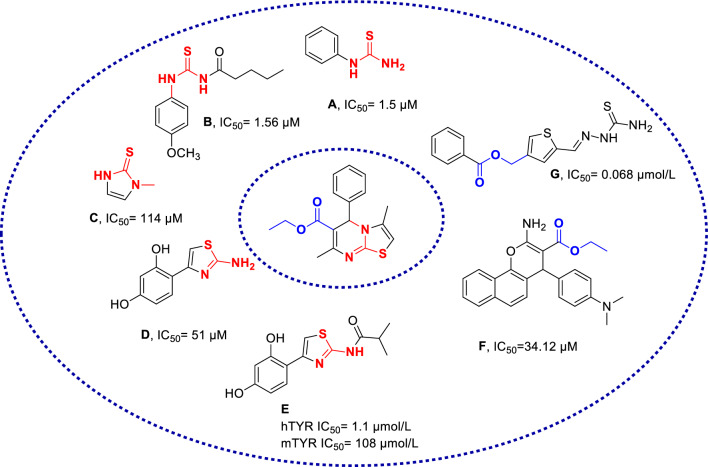
Previously reported tyrosinase inhibitor and newly designed compound

Tyrosinase inhibition with thiourea backbone and its derivatives showed promise results. Notably, phenylthiourea (compound **A**) has emerged as a potent tyrosinase inhibitor, and compound **B** with phenylthiourea structure, exhibited an impressive IC_50_ value of 5.4 µM [11]. Building on these encouraging findings, researchers have explored various derivatives of thiourea-based compounds, some of which are illustrated in Fig. 1. Another noteworthy example is methimazole (compound **C**), an approved drug with a thiourea structure, which has demonstrated inhibitory activity against tyrosinase [12]. Compound **D**, featuring a thiazole ring, has also exhibited strong tyrosinase inhibitory activity. Structure–activity relationship (SAR) studies have indicated that substituting the 2-amino group in the thiazole ring is pivotal for enhancing inhibitory potency. Modifications based on this structure have led to the development of Compound **E**, which shows improved tyrosinase inhibition [7]

Moreover, the incorporation of ethyl acetate functionality, as exemplified by compounds **F** and **G** [13, 14], appears to enhance the tyrosinase inhibitory potential of these derivatives significantly. This enhancement could potentially increase their ability to interact with critical residues at the enzyme's binding site.

While the tyrosinase inhibitory properties of thiazolopyrimidine compounds are limited, their unique structural features warrant exploration. Thiazolopyrimidine is a heterocyclic compound that combines the beneficial aspects of both thiazole and pyrimidine rings and a thiourea moiety embedded within its chemical framework. This combination of elements imparts diverse properties and suggests potential applications in pharmaceutical sciences and medicinal chemistry [15–17].

In summary, the design of tyrosinase inhibitors involves the strategic modification and incorporation of functional groups to optimize inhibitory potency. Exploring thiazolopyrimidine compounds adds a novel dimension to this research, offering the potential for multifaceted applications in medicinal chemistry [18].

Considering the biological properties of existing derivatives in Fig. 1 as tyrosinase inhibitors, a novel series of thiazolopyrimidine was synthesized to evaluate their tyrosinase inhibitory activity. Subsequently, SARs and kinetic studies were performed. Cavity detection was further executed to find a suitable site for noncompetitive inhibitors for in sillico assessments. Next, molecular docking and molecular dynamic studies were executed to properly evaluate the behavior of the most potent analog in the enzyme binding site.

## Results and discussion

### Chemistry

**Scheme 1:**
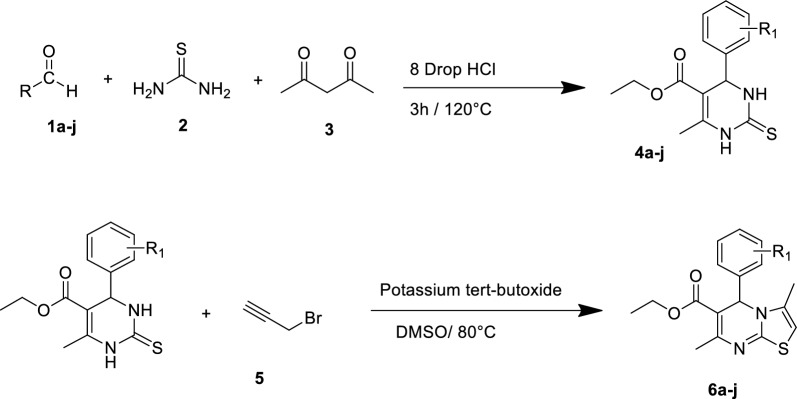
Synthesis of thiazolopyrimidine derivatives **6a**–**j**

A mixture of different aldehydes (**1a**–**j**), thiourea (**2**), ethyl acetoacetate (**3**) and catalytic amount of hydrochloric acid in acetic acid as solvent was heated under reflux for 3 h (Scheme 1). After the reaction was completed, the reaction mixture was poured into cold water to eliminate the byproducts. The precipitates were filtered off, washed with cold water, and purified using recrystallization from ethanol to synthesize **4a**–**j**. In the next step, **4a**–**j** were added to the DMSO charged with potassium tertiobutoxide and the reaction was sire for around 30 min at 80 ℃. Next, propargyl bromide (**5**) was added to the mixture [19]. The mixture was stirred at room temperature for an extra 5 h, poured into ice water, and then filtered to give the desired products **6a**–**j**.

### Tyrosinase inhibitory activity

In vitro tyrosinase inhibitory activity of synthesized compounds, **6a**–**j** was performed and compared with kojic acid as the reference inhibitor. The results of the anti-tyrosinase assay were presented in Table 1 in terms of IC_50_. In this series, all compounds except **6h** exhibited comparable inhibition against tyrosinase with IC_50_ values ranging from 28.50 to > 250 µM compared with a positive control with an IC_50_ value of 43.50 µM.

**Table 1:** Tyrosinase inhibitory activities of **6a**–**j**^a^


Compound	R	IC_50_ (µM)	SD (µM)
**6a**	H	28.50	4.8
**6b**	2-F	40.83	4.3
**6c**	3-F	56.00	8.0
**6d**	2-Cl	34.17	4.9
**6e**	4-Cl	37.67	6.0
**6f**	3-Br	85.33	8.8
**6g**	3-NO_2_	45.17	5.4
**6h**	4-Me	> 250	–
**6i**	4-OMe	35.67	6.6
**6j**	4-OH-3-OCH_3_	40.83	5.1
**Kojic acid**^b^	**–**	43.50	5.8

^a^Data presented here are the mean ± S.E of three independent experiments

^b^Positive control

The unsubstituted derivative **6a** demonstrated a strong inhibitory effect against tyrosinase, with an IC_50_ value of 28.50 μM. Next, different moieties were introduced at various positions of the phenyl pendant to assess the SAR. The evaluation of fluorine atoms, as small and strong electron-withdrawing halogen groups on the phenyl ring, showed that both **6b** and **6c** had reduced potency compared to the unsubstituted analog **6a**. Interestingly, *ortho*-fluorine analogs exhibited slightly better potency compared to *meta* analogs.

Replacing fluorine with a bulkier chlorine group resulted in improved potency in the 2-chloro substituent (**6d**, IC_50_ = 34.17 μM), followed by the 4-chloro substituent, **6e**, with an IC_50_ value of 37.67 μM. The 3-bromo derivative (**6f**) had inferior potency compared to the fluorine and chlorine-substituted analogs, with an IC_50_ value of 85.33 μM. Furthermore, introducing a nitro group at the *meta* position was evaluated (**6g**). Results showed that the presence of a strong electron-withdrawing group, capable of participating in hydrogen bonding interactions, demonstrated better activity compared to the 3-bromine moiety. However, this modification was still unsuccessful in improving the inhibition compared to the unsubstituted analog **6a**.

Subsequently, the inhibitory effect of electron-donating groups was assessed, revealing a complete loss of activity in the 4-methyl substitution (**6h**). Notably, the bioisosteric replacement of the methyl group with a methoxy group (**6i**) resulted in a 25-fold improvement in potency compared to compound **6h**, with an IC_50_ value of 35.67 μM. Additionally, the impact of methyl multi-substitutions was evaluated. The introduction of a 4-hydroxy-3-methoxy group (**6j**) slightly decreased the activity, with an IC_50_ value of 40.83 μM, compared to compound **6i**.

Overall, the results suggest that the unsubstituted analog was the most potent derivative in this set. The assessments of different substitutions revealed that the presence of a heteroatom capable of participating in hydrogen bonding interactions favored inhibition *vs* substituted derivatives. Furthermore, the evaluation of halogen groups demonstrated that chlorine substitutions exhibited good potency among **6b**–**f**. A moderate level of lipophilicity was advantageous for inhibition compared to other substituted derivatives. These findings highlight the significance of specific substitutions and their impact on the inhibitory potency against tyrosinase.

### Enzyme kinetic studies

**Fig. 2:**
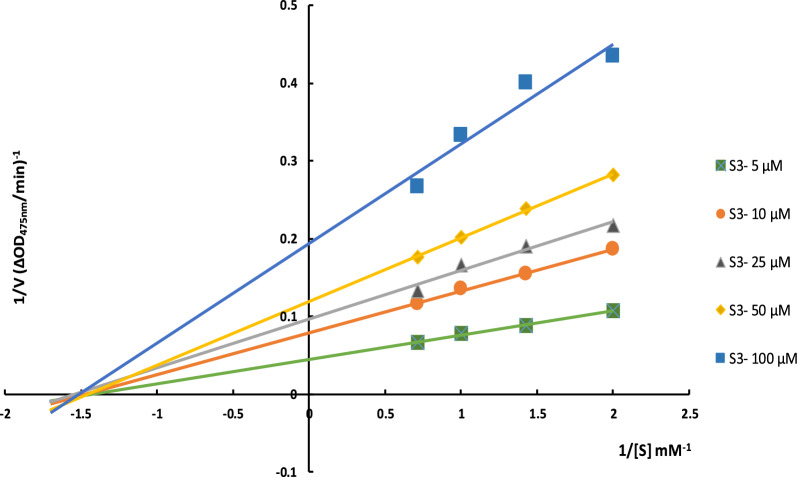
Lineweaver–Burk plot for the inhibition of tyrosinase-catalyzed L-Dopa oxidation by **6a**

Next, the kinetic assessment of **6a** as the most potent analog was examined to determine the type of inhibition (Fig. 2). Lineweaver–Burk plots (plot of 1/V versus 1/[S]) for the inhibition of tyrosinase was obtained with several concentrations of **6a** (as the inhibitor) and L-Dopa (as the substrate). As can be seen in Table 2, as the inhibitor concentration raised, the value of *V*_*max*_ was reduced, but *Km* was not affected by the concentration, confirming noncompetitive inhibitor.

**Table 2:** Kinetic parameters for the compounds **6a** against tyrosinase

Concentration (µM)	*V*_*max*_(mM/Min)	*K*_*m*_ (mM)
5	22.22	0.69
10	12.66	0.68
25	10.35	0.65
50	8.40	0.69
100	5.15	0.66

### Effect of compound 6a on UV/VIS Spectra of tyrosinase

**Fig. 3:**
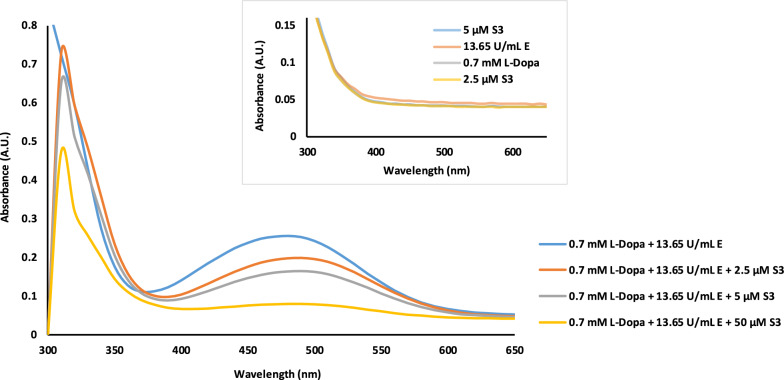
UV/VIS spectra for the interaction between tyrosinase and compound **6a**

UV spectra of compound **6a,** tyrosinase and L-Dopa were analyzed separately. As shown in Fig. 3, no significant absorbance was observed at 475 nm, which is the wavelength used to determine tyrosinase inhibition. However, when L-Dopa was added to tyrosinase without an inhibitor, the highest absorbance was observed, confirming the generation of dopachrome. The absorbance intensity decreased dose-dependently after adding compound **6a** at various concentrations. This observation confirms the inhibitory potency of **6a** against tyrosinase and its ability to prevent dopachrome formation.

### Molecular docking

**Fig. 4:**
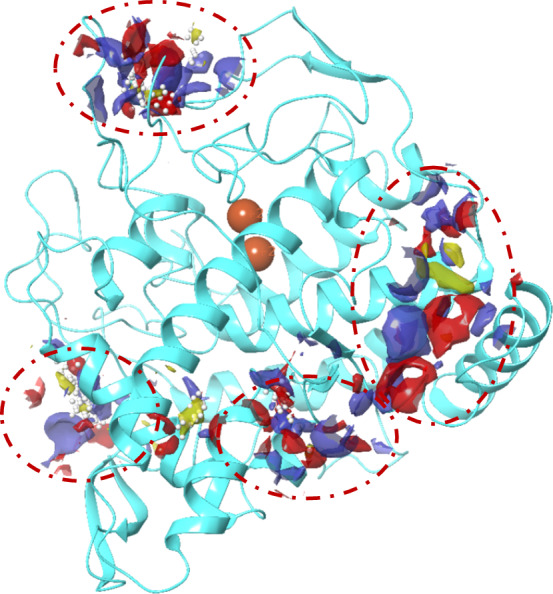
Potential binding sites for tyrosinase noncompetitive inhibitor colored with red dash line

A molecular docking study investigated the crucial interactions between compound **6a** and tyrosinase. The objective was to gain insights into the binding mode and potential key interactions between the compound and the enzyme. First, cavity detection was applied to find the new allosteric site of the enzyme. As exhibited in Fig. 4, four sites were determined regardless of the tyrosinase active site. The results of the site and druggability score of potential binding sites are presented in Table 3.

**Table 3:** Site core and druggability score of potential binding sites of tyrosinase enzyme

Potential binding sites	Site score	Druggability score
Site 1	0.779	0.753
Site 2	0.808	0.823
Site 3	0.975	0.910
Site 4	0.627	0.593

Next, **6a,** as the potent inhibitor, was docked on all four potential binding sites of the enzyme. The results of the docking score for each site need and binding free energies calculated using MM-GBSA are exhibited in Table 4.

**Table 4:** Docking score and MM-GBSA of **6a** as the different potential binding sites

Potential binding sites	Docking Score	MM-GBSA
Site 1	− 4.574	− 21.43
Site 2	− 5.803	− 23.48
Site 3	− 7.389	− 30.38
Site 4	− 4.378	− 20.08

**Fig. 5:**
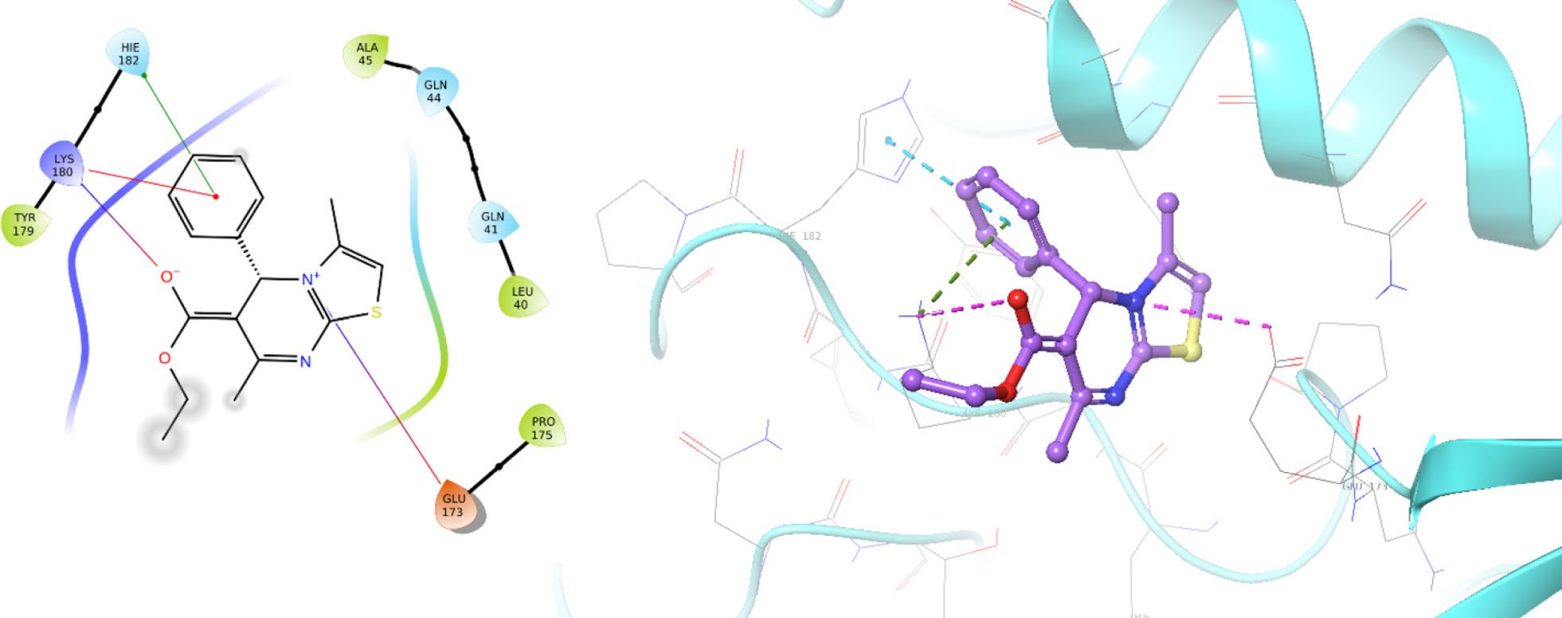
The binding mode of compound **6a** within the allosteric site of mushroom tyrosinase (PDB ID: 2Y9X)

Considering the docking score, interactions, and MM-GBSA, site 3 showed the highest affinity in comparison to other identified sites (Fig. 4). This site was also presented as an allosteric site in the previous study using different software in which His178, Lys 180, and Gln44 were categorized as important residues of the allosteric site [20]. As exhibited in Fig. 5, the phenyl ring exhibited pi-cation and pi-pi stacking interactions with Lys180 and His182. The thiazolopyrimidine ring revealed a salt bridge interaction with Glu173, and an oxygen atom of ethyl acetate also recorded another salt bridge interaction with Lys180.

This investigation revealed that these derivatives function as non-competitive inhibitors, binding to an allosteric site. The results of the molecular docking study for all derivatives have been included in the additional file 1: Table S1). All derivatives exhibited the same binding mode and interacted with Lys180 with docking score values of − 4.497 to − 7.389. It is evident that these derivatives attach to the enzyme's surface, and docking studies have further indicated that increased branching and substitution may impede access to the anchoring site, thereby hindering interactions with the enzyme's binding site. This finding offers valuable insights into the decreased potency observed in the substituted analogs, **6b**–**j**.

### Molecular dynamic simulations

**Fig. 6:**
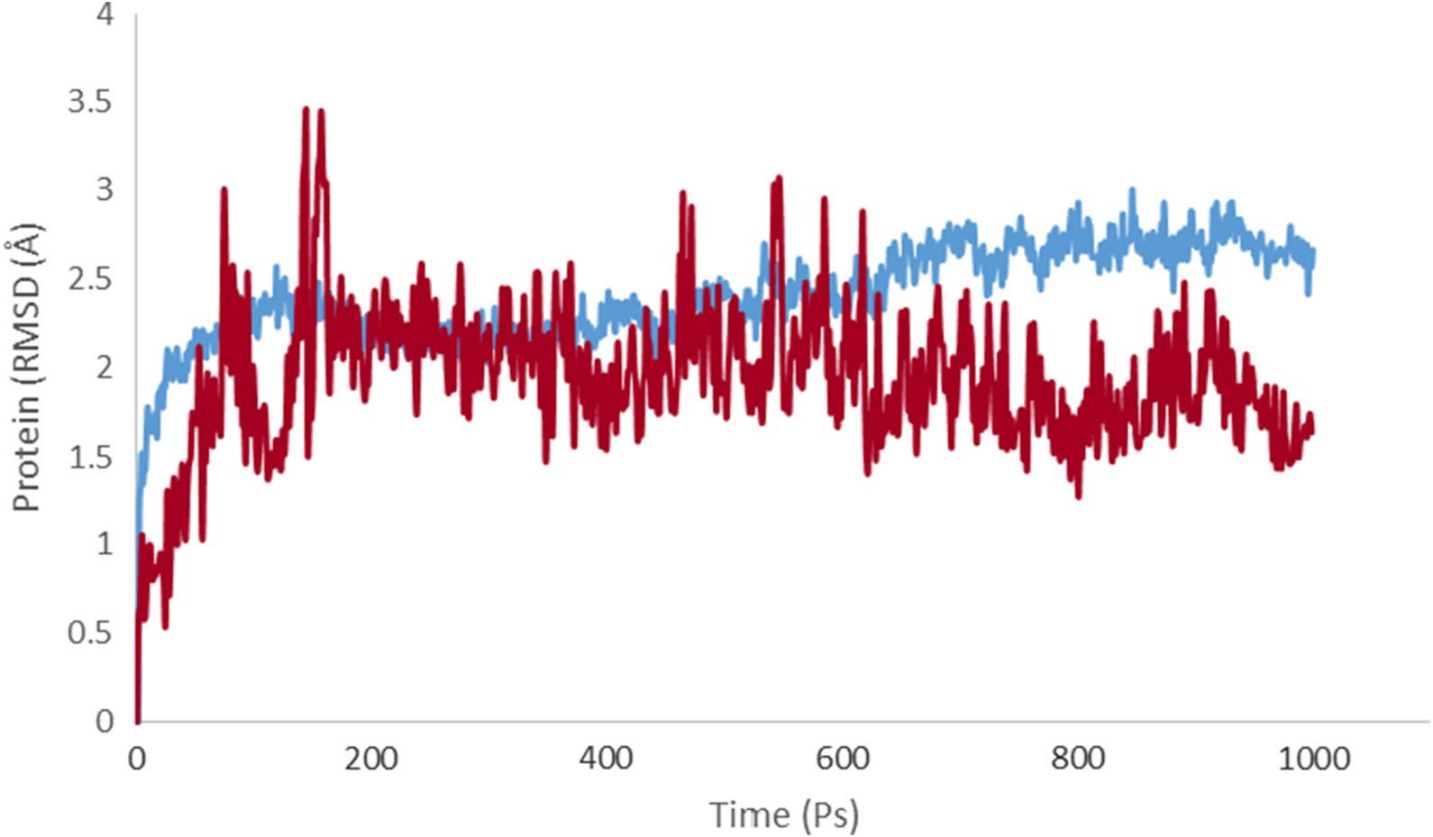
The RMSD values of the tyrosinase (blue) and **6a**-tyrosinase complex (red) over the simulation period

Molecular dynamics (MD) simulations were conducted to assess the behavior and stability of the **6a-**tyrosinase complex compared to the tyrosinase enzyme. The backbone root mean square deviation (RMSD) throughout the MD simulation is depicted in Fig. 6. In the simulation's initial phase, the apoenzyme's RMSD value exhibited a gradual increase (0–50 ps) before stabilizing at an average value of 2.2 Å. Subsequently, there was a slight increase to 2.5 Å towards the end of the simulation period.

In contrast, the RMSD value of the **6a**-tyrosinase complex displayed some fluctuations during the first 200 ps, followed by a steady decrease with lower RMSD values compared to the enzyme alone throughout the entire simulation. This suggests that the presence of compound **6a** contributed to enhancing the stability of the enzyme.

**Fig. 7:**
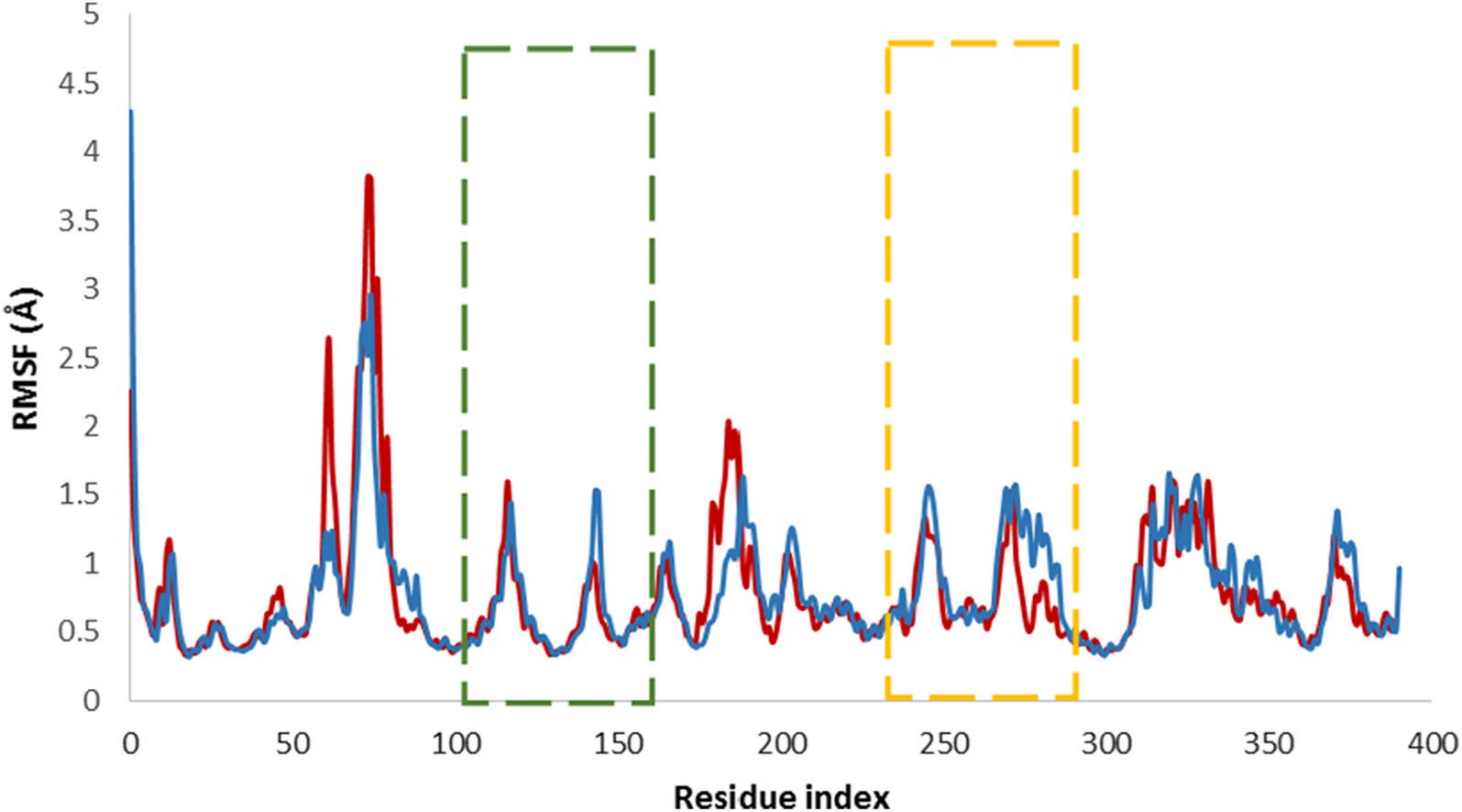
The RMSF values of the tyrosinase (blue) and **6a**-tyrosinase complex (red) over the simulation period

The detailed mechanism of ligand interactions with the enzyme was investigated using root mean square fluctuations (RMSF). The ligand's binding to the tyrosinase decreased the movement of residues, primarily due to non-bonding interactions between the ligand and the enzyme. The high fluctuations observed in the RMSD values can be attributed to the unstructured region of the enzyme, specifically the residues between Ile60 and Val88, as well as the N-terminal of the enzyme (Fig. 7). As indicated, the reduction in movement in the region spanning residues 107 to 150 (highlighted by the green dashed line), along with residues 239 to 291 (highlighted by the orange dashed line), compared to the apoenzyme in these areas, played a significant role in stabilizing this allosteric site region.

**Fig. 8:**
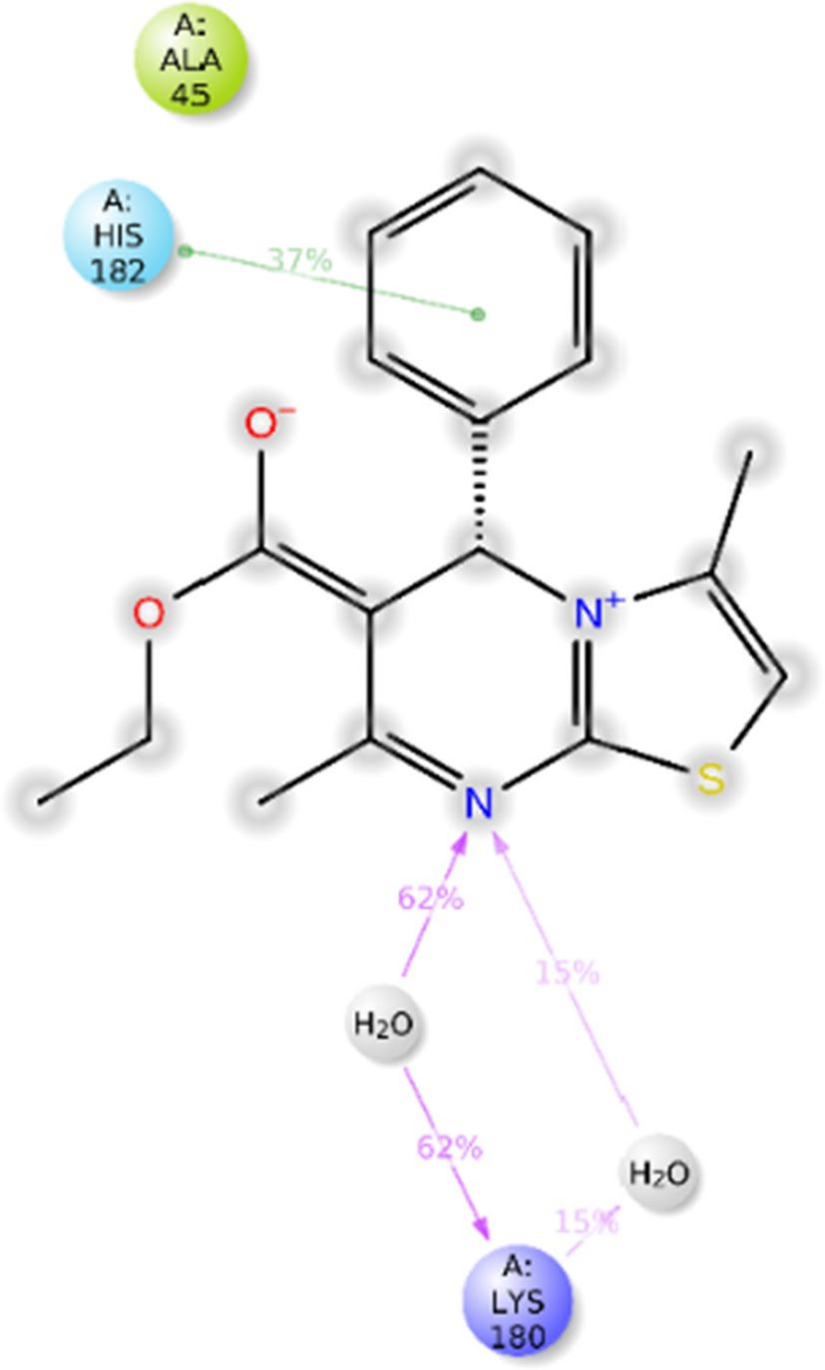
2D interaction pattern of **6a** within the binding site of tyrosinase over 15% of simulation time

The analysis unveiled significant interactions between compound **6a** and the enzyme's binding site. One noteworthy interaction involved hydrogen bonding between the thiazolopyrimidine group of compound **6a** and the Lys180 residue, mediated by water within the binding pocket. This interaction was crucial in stabilizing the compound within the binding site. Furthermore, a pi-pi stacking interaction was observed between the phenyl moiety of compound **6a** and His182, a pivotal residue of the tyrosinase enzyme. This interaction further bolstered the binding affinity and stability of the compound within the site (Fig. 8).

These interactions between compound **6a** and the enzyme’s binding pocket provide valuable insights into the molecular basis of its inhibitory activity against the enzyme. They highlight key molecular interactions contributing to its binding and stability within the active site.

## Conclusion

In summary, a novel class of thiazolopyrimidine-based tyrosinase inhibitors was successfully developed. The structures of all synthesized derivatives were confirmed using various spectroscopy techniques, including FTIR, Mass, ^1^H-NMR, and ^13^C-NMR. Among these derivatives, compound **6a**, an unsubstituted derivative, demonstrated the highest potency with an IC_50_ value of 28.50 µM, compared with the reference inhibitor kojic acid (IC_50_ = 43.50 µM). Kinetic evaluation of compound **6a** revealed a noncompetitive mode of inhibition. UV/VIS spectra analysis further supported the inhibitory effect of compound **6a** on tyrosinase activity. The compound effectively prevented the conversion of L-Dopa into dopachrome in a dose-dependent manner. Moreover, MD simulations were performed to study the dynamic behavior and stability of the **6a**-tyrosinase complex. The simulations revealed significant interactions between compound **6a** and the binding site of tyrosinase.

Overall, this study presents the novel class of thiazolopyrimidine derivatives as promising tyrosinase inhibitors, supported by both experimental and computational analyses.

## Material and methods

### Chemistry

The synthetic method followed according to the previously reported procedures [19]. Briefly, 10 mmol of different aldehydes (**1a**–**j**), 15 mmol of thiourea (**2**), 10 mmol of ethyl acetoacetate (**3**) and catalytic amount of hydrochloric acid in acetic acid as solvent was heated under reflux for 3 h. After the reaction was completed, the reaction mixture was poured into cold water to eliminate the byproducts. The precipitates were filtered off, washed with cold water, and purified using recrystallization from ethanol to synthesize 4a-j. In the next step, **4a**–**j** were added to the DMSO charged with potassium tertiobutoxide and the reaction was sire for around 30 min at 80˚C. Next, propargyl bromide (**5**) was added to the mixture. The mixture was stirred at room temperature for an extra 5 h, poured into ice water, and then filtered to give the desired products **6a**–**j**.

#### Ethyl 3,7-dimethyl-5-phenyl-5H-thiazolo[3,2-a]pyrimidine-6-carboxylate (6a)

Yield: 69%. MP: 110–111 °C. White powder. IR (KBr): 3109, 1732, 1540, 1482, 1263 cm^−1^. ^1^H NMR (400 MHz, DMSO-*d*_6_) δ 7.42 (t, *J* = 8.6 Hz, 2H), 7.29 (t, *J* = 8.0 Hz, 1H), 7.22 (d, *J* = 8.2 Hz, 2H), 6.39 (d, *J* = 1.5 Hz, 1H), 6.11 (s, 1H), 4.16–4.13 (m, 2H), 2.29 (s, 3H), 2.05 (d, *J* = 1.3 Hz, 3H), 1.09 (t, *J* = 7.1 Hz, 3H). ^13^C NMR (100 MHz, DMSO-*d*_6_) δ 166.25, 166.22, 158.99, 143.82, 135.67, 128.95, 127.42, 126.22, 101.08, 99.67, 57.77, 54.74, 22.88, 14.39, 13.41. *Anal*. Calcd for C_17_H_18_N_2_O_2_S: C 64.94; H 5.77; N 8.91; S 10.20; Found: C 64.83; H 5.96; N 9.11; S 10.35.

#### Ethyl 5-(2-fluorophenyl)-3,7-dimethyl-5*H*-thiazolo[3,2-*a*]pyrimidine-6-carboxylate (6b)

Yield: 65%. MP: 111–112 °C. White powder. IR (KBr): 3089, 1733, 1547, 1456, 1231, 1001 cm^−1^. ^1^H NMR (400 MHz, DMSO-*d*_6_) δ 7.43 (td, *J* = 7.8, 1.9 Hz, 1H), 7.39–7.29 (m, 1H), 7.24–7.13 (m, 2H), 6.48–6.38 (m, 2H), 4.03–3.88 (m, 2H), 2.27 (s, 3H), 2.04 (d, *J* = 1.3 Hz, 3H), 1.11 (t, *J* = 7.1 Hz, 3H). ^13^C NMR (100 MHz, DMSO-*d*_6_) δ 166.36, 165.83, 159.54 (^1^*J*_C–F_ = 246 Hz), 156.21, 135.89, 131.13 (^2^*J*_C–F_ = 7 Hz), 131.00 (^4^*J*_C–F_ = 2 Hz), 130.29 (^3^*J*_C–F_ = 4 Hz), 125.66 (^3^*J*_C–F_ = 4 Hz), 116.12 (^2^*J*_C–F_ = 22 Hz), 101.33, 98.56, 59.49, 51.71 (^3^*J*_C–F_ = 2 Hz), 23.85, 14.44, 13.51 (*J*_C–F_ =  = 2 Hz). *Anal*. Calcd for C_17_H_17_FN_2_O_2_S: C 61.43; H 5.16; N 8.43; S 9.65; Found: C 61.31; H 5.36; N 8.21; S 9.41.

#### Ethyl 5-(3-fluorophenyl)-3,7-dimethyl-5*H*-thiazolo[3,2-*a*]pyrimidine-6-carboxylate (6c)

Yield: 70%. MP: 113–114 °C. White powder. IR (KBr): 3112, 1737, 1556, 1461, 1218, 994 cm^−1^. ^1^H NMR (400 MHz, DMSO-*d*_6_) δ 7.40 (td, *J* = 8.0, 6.0 Hz, 1H), 7.17–6.99 (m, 3H), 6.50 (d, *J* = 1.5 Hz, 1H), 6.20 (s, 1H), 4.12–3.97 (m, 2H), 2.24 (s, 3H), 2.04 (d, *J* = 1.3 Hz, 3H), 1.19 (t, *J* = 7.1 Hz, 3H). ^13^C NMR (100 MHz, DMSO-*d*_6_) δ 166.51, 166.07, 163.60 (^1^*J*_C–F_ = 243 Hz), 156.02, 146.39 (^3^*J*_C–F_ = 6 Hz), 135.87, 131.51 (^3^*J*_C–F_ = 8 Hz), 122.71 (^4^*J*_C–F_ = 3 Hz), 115.60 (^2^*J*_C–F_ = 21 Hz), 113.57 (^2^*J*_C–F_ = 22 Hz), 101.85, 99.35, 59.72, 56.81, 56.79, 23.98, 14.66, 13.78. *Anal*. Calcd for C_17_H_17_FN_2_O_2_S: C 61.43; H 5.16; N 8.43; S 9.65; Found: C 61.27; H 5.33; N 8.61; S 9.84.

#### Ethyl 5-(2-chlorophenyl)-3,7-dimethyl-5*H*-thiazolo[3,2-*a*]pyrimidine-6-carboxylate (6d)

Yield: 64%. MP: 114–115 °C. White powder. IR (KBr): 3080, 1734, 1531, 1428, 1257, 739 cm^−1^. ^1^H NMR (400 MHz, DMSO-*d*_6_) δ 7.55 (dd, *J* = 7.6, 1.8 Hz, 1H), 7.41 (dd, *J* = 7.7, 1.6 Hz, 1H), 7.33 (dtd, *J* = 19.5, 7.4, 1.7 Hz, 3H), 6.54 (s, 1H), 6.41 (d, *J* = 1.5 Hz, 1H), 3.99 (qt, *J* = 7.0, 3.5 Hz, 2H), 2.27 (s, 3H), 2.05 (d, *J* = 1.3 Hz, 3H), 1.11 (t, *J* = 7.1 Hz, 3H). ^13^C NMR (100 MHz, DMSO-*d*_6_) δ 166.55, 165.94, 156.06, 141.71, 136.22, 131.06, 130.53, 130.16, 129.81, 128.72, 101.23, 99.23, 59.44, 55.07, 24.02, 14.73, 14.34. *Anal*. Calcd for C_17_H_17_ClN_2_O_2_S: C 58.53; H 4.91; N 8.03; S 9.19; Found: C 58.30; H 5.12; N 7.88; S 9.05.

#### Ethyl 5-(4-chlorophenyl)-3,7-dimethyl-5H-thiazolo[3,2-a]pyrimidine-6-carboxylate (6e)

Yield: 78%. MP: 113–114 °C. White powder. IR (KBr): 3099, 1731, 1569, 1471, 1224, 747 cm^−1^. ^1^H NMR (400 MHz, DMSO-*d*_6_) δ 7.40 (d, *J* = 8.4 Hz, 2H), 7.25 (d, *J* = 8.5 Hz, 2H), 6.51 (d, *J* = 1.5 Hz, 1H), 6.17 (s, 1H), 4.01 (dq, *J* = 21.0, 7.1 Hz, 2H), 2.25 (s, 3H), 2.03 (d, *J* = 1.4 Hz, 3H), 1.10 (t, *J* = 7.1 Hz, 3H). ^13^C NMR (100 MHz, DMSO-*d*_6_) δ 165.99, 165.65, 152.39, 142.64, 135.94, 132.23, 129.25, 128.73, 102.03, 99.99, 59.73, 56.80, 23.79, 14.66, 13.77. *Anal*. Calcd for C_17_H_17_ClN_2_O_2_S: C 58.53; H 4.91; N 8.03; S 9.19; Found: C 58.28; H 4.76; N 7.85; S 9.42.

#### Ethyl 5-(3-bromophenyl)-3,7-dimethyl-5*H*-thiazolo[3,2-*a*]pyrimidine-6-carboxylate (6f)

Yield: 68%. MP: 112–113 °C. White powder. IR (KBr): 3110, 1735, 1552, 1475, 1249, 1023 cm^−1^. ^1^H NMR (400 MHz, DMSO-*d*_6_) δ 7.53–7.46 (m, 1H), 7.43 (t, *J* = 1.9 Hz, 1H), 7.32 (t, *J* = 7.8 Hz, 1H), 7.23 (dt, *J* = 7.8, 1.4 Hz, 1H), 6.50 (d, *J* = 1.5 Hz, 1H), 6.17 (s, 1H), 4.17–3.94 (m, 2H), 2.24 (s, 3H), 2.03 (d, *J* = 1.3 Hz, 3H), 1.20 (t, *J* = 7.1 Hz, 3H). ^13^C NMR (100 MHz, DMSO-*d*_6_) δ 166.55, 166.00, 156.16, 146.22, 135.84, 131.68, 131.51, 129.41, 125.77, 122.11, 101.88, 99.27, 59.72, 56.80, 24.01, 14.64, 13.83. *Anal*. Calcd for C_17_H_17_BrN_2_O_2_S: C 51.92; H 4.36; N 7.12; S 8.15; Found: C 51.77; H 3.47; N 7.26; S 8.01.

#### Ethyl 3,7-dimethyl-5-(3-nitrophenyl)-5*H*-thiazolo[3,2-*a*]pyrimidine-6-carboxylate (6g)

Yield: 82%. MP: 151–152 °C. Light brown powder. IR (KBr): 3110, 1732, 1551, 1348, 1205, 1077 cm^−1^. ^1^H NMR (400 MHz, DMSO-*d*_6_) δ 8.15 (dt, *J* = 5.5, 2.8 Hz, 1H), 8.12–8.06 (m, 1H), 7.73–7.59 (m, 2H), 6.52 (d, *J* = 1.5 Hz, 1H), 6.35 (s, 1H), 4.13–3.95 (m, 2H), 2.25 (s, 3H), 2.04 (d, *J* = 1.4 Hz, 3H), 1.20 (t, *J* = 7.1 Hz, 3H). ^13^C NMR (100 MHz, DMSO-*d*_6_) δ 166.70, 165.92, 156.67, 148.18, 145.57, 135.80, 133.32, 131.15, 123.62, 121.39, 102.07, 98.94, 59.81, 56.77, 24.09, 14.58, 13.83. *Anal*. Calcd for C_17_H_17_N_3_O_4_S: C 56.81; H 4.77; N 11.69; S 8.92; Found: C 56.64; H 4.55; N 11.87; S 8.73.

#### Ethyl 3,7-dimethyl-5-(p-tolyl)-5*H*-thiazolo[3,2-*a*]pyrimidine-6-carboxylate (6h)

Yield: 71%. MP: 114–115 °C. White powder. IR (KBr): 3107, 1736, 1536, 1469, 1244, 1005 cm^−1^. ^1^H NMR (400 MHz, DMSO-*d*_6_) δ 7.19–7.07 (m, 4H), 6.44 (d, *J* = 1.5 Hz, 1H), 6.10 (s, 1H), 4.02 (dddd, *J* = 17.9, 10.9, 7.1, 3.8 Hz, 2H), 2.24 (s, 3H), 2.24 (s, 3H), 2.02 (s, 3H), 1.19 (t, *J* = 7.1 Hz, 3H). ^13^C NMR (100 MHz, DMSO-*d*_6_) δ 166.34, 166.17, 155.39, 141.10, 137.95, 135.95, 129.67, 126.65, 101.44, 99.99, 59.57, 57.09, 23.90, 21.12, 14.68, 13.79. *Anal*. Calcd for C_18_H_20_N_2_O_2_S: C 65.83; H 6.14; N 8.53; S 9.76; Found: C 65.63; H 6.29; N 8.72; S 9.58.

#### Ethyl 5-(4-methoxyphenyl)-3,7-dimethyl-5*H*-thiazolo[3,2-*a*]pyrimidine-6-carboxylate (6i)

Yield: 72%. MP: 111–112 °C. White powder. IR (KBr): 3104, 1734, 1537, 1438, 1279 cm^−1^. ^1^H NMR (400 MHz, DMSO-*d*_6_) δ 7.18 (d, *J* = 8.6 Hz, 2H), 6.88 (d, *J* = 8.6 Hz, 2H), 6.44 (d, *J* = 1.5 Hz, 1H), 6.08 (s, 1H), 4.09–3.93 (m, 2H), 3.71 (s, 3H), 2.24 (s, 3H), 2.03 (d, *J* = 1.3 Hz, 3H), 1.18 (t, *J* = 7.1 Hz, 3H). ^13^C NMR (100 MHz, DMSO-*d*_6_) δ 166.20, 165.95, 159.39, 155.21, 136.28, 135.98, 128.07, 114.42, 101.41, 100.17, 59.56, 56.80, 55.52, 23.90, 14.69, 13.82. *Anal*. Calcd for C_18_H_20_N_2_O_3_S: C 62.77; H 5.85; N 8.13; S 9.31; Found: C 62.54; H 5.99; N 8.27; S 9.58.

#### Ethyl 5-(4-hydroxy-3-methoxyphenyl)-3,7-dimethyl-5*H*-thiazolo[3,2-*a*]pyrimidine-6-carboxylate (6j)

Yield: 77%. MP: 121–122 °C. White powder. IR (KBr): 3532, 1735, 1566, 1439, 1222, 1091 cm^−1^. ^1^H NMR (400 MHz, DMSO-*d*_6_) δ 9.13 (s, 1H), 6.81 (d, *J* = 2.2 Hz, 1H), 6.71 (d, *J* = 8.1 Hz, 1H), 6.62 (dd, *J* = 8.2, 2.0 Hz, 1H), 6.44 (d, *J* = 1.6 Hz, 1H), 6.03 (s, 1H), 4.12–3.97 (m, 2H), 3.70 (s, 3H), 2.24 (s, 3H), 2.11–2.00 (m, 3H), 1.20 (t, *J* = 7.1 Hz, 3H). ^13^C NMR (100 MHz, DMSO-*d*_6_) δ 166.31, 166.22, 155.18, 147.67, 146.93, 136.10, 135.19, 119.24, 116.02, 110.88, 101.25, 100.19, 59.54, 57.05, 55.98, 23.87, 14.75, 13.86. *Anal*. Calcd for C_18_H_20_N_2_O_4_S: C 59.98; H 5.59; N 7.77; S 8.89; Found: C 59.86; H 5.74; N 7.93; S 8.66.

### Tyrosinase inhibitory assay

The tyrosinase inhibitory activities of derivatives were performed according to the previously reported procedures. All the test samples were first dissolved in DMSO at dilution to the required final concentrations. Initially, in a 96-well microplate, 10 µl of test samples were added to 160 µl of phosphate buffer (pH = 6.8), and then 10 µl tyrosinase (EC 1.14.18.1; 500 U/ml) was added. After the mixture was pre-incubated at 28 °C for 20 min, 20 µl of L-Dopa solution (7 mM) was added to the mixture. DMSO without test compounds was used as the control, and kojic acid was used as a positive control. After 5 min incubation absorbance of samples was measured at 490 nm. Each assay was conducted as three separate replicates. The inhibitory activity of the tested compounds was expressed as the concentration that inhibited 50% of the enzyme activity (IC_50_).

### Enzyme kinetic studies

The kinetic study for tyrosinase inhibition by **6a** as the most potent analog was carried out using four different concentrations of inhibitor (5, 10, 25, 50, and 100 µM) against tyrosinase with different concentrations of L-Dopa (0.25, 0.5, 0.75, and 1 mM) as the substrate. The Lineweaver–Burk reciprocal plot was provided by plotting 1/V against 1/[S] at variable concentrations of the L-Dopa.

### Molecular docking

The molecular docking studies were performed using the Maestro Molecular Modeling platform of Schrödinger. The 3D crystal structure of tyrosinase was retrieved from the Protein Data Bank (PDB code: 2Y9X). Protein was prepared in which the water molecules and the cognate ligand (tropolone) were removed from the receptor and, the hydrogen atoms were added and non-polar hydrogens were merged into related atoms of the receptor via protein preparation. To prepare the ligand, the 2D structures of the ligands were drawn in ChemDraw, converted into SDF files, and subjected to the ligprep module. Ligands were prepared by OPLS_2005 force field using EPIK. To find the possible allosteric site, site map tools were applied to find five possible sites. And all parameters were set as default. The grid box was generated for each binding site with a box size of 20 Å; the derivative was docked on binding sites using induced-fit docking, reporting 10 poses per ligand to form the final complex.

### MD simulation

The molecular simulation was conducted utilizing the Desmond of Schrödinger package. To prepare the system for MD simulation, the protein–ligand complexes were immersed in an orthorhombic box of suitable dimensions with periodic boundary conditions and solvated using explicit water molecules of the SPC type. Additionally, the system was neutralized by incorporating an appropriate number of counter-ions, and a 0.15 M solution of NaCl was employed to mimic realistic cellular ionic concentrations. The MD protocol involved minimization, pre-production, and production MD simulation steps. Finally, the system was subjected to produce MD simulations for 100 ns for ao enzyme andprotein–ligand complex. The dynamic behavior and structural changes of the systems were analyzed by the calculation of the RMSD and RMSF.

### Supplementary Information

**Additional file 1: Fig. S1.** ^1^H NMR (400 MHz, DMSO-*d*_*6*_); Ethyl 3,7-dimethyl-5-phenyl-5H-thiazolo[3,2-a]pyrimidine-6-carboxylate (**6a**). **Fig. S2.** ^13^C NMR (100 MHz, DMSO-d_6_); Ethyl 3,7-dimethyl-5-phenyl-5H-thiazolo[3,2-a]pyrimidine-6-carboxylate (**6a**). **Fig. S3.** ^1^H NMR (400 MHz, DMSO-d_6_); Ethyl 5-(2-fluorophenyl)-3,7-dimethyl-5H-thiazolo[3,2-a]pyrimidine-6-carboxylate (**6b**). **Fig. S4.** ^13^C NMR (100 MHz, DMSO-d_6_); Ethyl 5-(2-fluorophenyl)-3,7-dimethyl-5H-thiazolo[3,2-a]pyrimidine-6-carboxylate (**6b**). **Fig. S5.** ^1^H NMR (400 MHz, DMSO-d_6_); Ethyl 5-(3-fluorophenyl)-3,7-dimethyl-5H-thiazolo[3,2-a]pyrimidine-6-carboxylate (**6c**). **Fig. S6.** ^13^C NMR (100 MHz, DMSO-d_6_); Ethyl 5-(3-fluorophenyl)-3,7-dimethyl-5H-thiazolo[3,2-a]pyrimidine-6-carboxylate (**6c**). **Fig. S7.** ^1^H NMR (400 MHz, DMSO-*d*_*6*_*)*; Ethyl 5-(2-chlorophenyl)-3,7-dimethyl-5*H*-thiazolo[3,2-*a*]pyrimidine-6-carboxylate (**6d**). **Fig. S8*.*** ^13^C NMR (100 MHz, DMSO-*d*_*6*_); Ethyl 5-(2-chlorophenyl)-3,7-dimethyl-5*H*-thiazolo[3,2-*a*]pyrimidine-6-carboxylate (**6d**). **Fig. S9.** ^1^H NMR (400 MHz, DMSO-*d*_*6*_); Ethyl 5-(4-chlorophenyl)-3,7-dimethyl-5H-thiazolo[3,2-a]pyrimidine-6-carboxylate (**6e**). **Fig. S10*.*** ^13^C NMR (100 MHz, DMSO-*d*_*6*_)*;* Ethyl 5-(4-chlorophenyl)-3,7-dimethyl-5H-thiazolo[3,2-a]pyrimidine-6-carboxylate (**6e**). **Fig. S11.** ^1^H NMR (400 MHz, DMSO-*d*_*6*_); Ethyl 5-(3-bromophenyl)-3,7-dimethyl-5H-thiazolo[3,2-a]pyrimidine-6-carboxylate (**6f**). **Fig. S12*.*** ^13^C NMR (100 MHz, DMSO-*d*_*6*_*);* Ethyl 5-(3-bromophenyl)-3,7-dimethyl-5H-thiazolo[3,2-a]pyrimidine-6-carboxylate (**6f**). **Fig. S13.** ^1^H NMR (400 MHz, DMSO-*d*_*6*_); Ethyl 3,7-dimethyl-5-(3-nitrophenyl)-5H-thiazolo[3,2-a]pyrimidine-6-carboxylate (**6g**). **Fig. S14*.*** ^13^C NMR (100 MHz, DMSO-*d*_*6*_*)*; Ethyl 3,7-dimethyl-5-(3-nitrophenyl)-5*H*-thiazolo[3,2-*a*]pyrimidine-6-carboxylate (**6g**). **Fig. S15.** ^1^H NMR (400 MHz, DMSO-*d*_*6*_); Ethyl 3,7-dimethyl-5-(p-tolyl)-5H-thiazolo[3,2-a]pyrimidine-6-carboxylate (**6h**). **Fig. S16.** ^13^C NMR (100 MHz, DMSO-*d*_*6*_); Ethyl 3,7-dimethyl-5-(p-tolyl)-5H-thiazolo[3,2-a]pyrimidine-6-carboxylate (**6h**). **Fig. S17.** ^1^H NMR (400 MHz, DMSO-*d*_*6*_); Ethyl 5-(4-methoxyphenyl)-3,7-dimethyl-5H-thiazolo[3,2-a]pyrimidine-6-carboxylate (**6i**). **Fig. S18.** ^13^C NMR (100 MHz, DMSO-*d*_*6*_); Ethyl 5-(4-methoxyphenyl)-3,7-dimethyl-5H-thiazolo[3,2-a]pyrimidine-6-carboxylate (**6i**). **Fig. S19.** ^1^H NMR (400 MHz, DMSO-*d*_*6*_); Ethyl 5-(4-hydroxy-3-methoxyphenyl)-3,7-dimethyl-5H-thiazolo[3,2-a]pyrimidine-6-carboxylate (**6j**). **Fig. S20.** ^13^C NMR (100 MHz, DMSO-*d*_*6*_); Ethyl 5-(4-hydroxy-3-methoxyphenyl)-3,7-dimethyl-5H-thiazolo[3,2-a]pyrimidine-6-carboxylate (**6j**). **Table S1.** Results of molecular docking study of **6a**–**j** against tyrosinase binding site.

## Data Availability

The datasets generated and/or analysed during the current study are available in the Worldwide Protein Data Bank (wwPDB) repository. (https://www.rcsb.org/structure/2y9x).
