# Supplementary material for: Thiazolopyrimidine derivatives as novel class of small molecule tyrosinase inhibitor
Source: BMC Chem. 2023 Nov 19;17(1):156. doi: 10.1186/s13065-023-01077-z (PMC10658973; doi:10.1186/s13065-023-01077-z)
Supplement: Supplementary file 1 — Additional file 1: Fig. S1. 1H NMR (400 MHz, DMSO-d6); Ethyl 3,7-dimethyl-5-phenyl-5H-thiazolo[3,2-a]pyrimidine-6-carboxylate (6a). Fig. S2. 13C NMR (100 MHz, DMSO-d6); Ethyl 3,7-dimethyl-5-phenyl-5H-thiazolo[3,2-a]pyrimidine-6-carboxylate (6a). Fig. S3. 1H NMR (400 MHz, DMSO-d6); Ethyl 5-(2-fluorophenyl)-3,7-dimethyl-5H-thiazolo[3,2-a]pyrimidine-6-carboxylate (6b). Fig. S4. 13C NMR (100 MHz, DMSO-d6); Ethyl 5-(2-fluorophenyl)-3,7-dimethyl-5H-thiazolo[3,2-a]pyrimidine-6-carboxylate (6b). Fig. S5. 1H NMR (400 MHz, DMSO-d6); Ethyl 5-(3-fluorophenyl)-3,7-dimethyl-5H-thiazolo[3,2-a]pyrimidine-6-carboxylate (6c). Fig. S6. 13C NMR (100 MHz, DMSO-d6); Ethyl 5-(3-fluorophenyl)-3,7-dimethyl-5H-thiazolo[3,2-a]pyrimidine-6-carboxylate (6c). Fig. S7. 1H NMR (400 MHz, DMSO-d6); Ethyl 5-(2-chlorophenyl)-3,7-dimethyl-5H-thiazolo[3,2-a]pyrimidine-6-carboxylate (6d). Fig. S8. 13C NMR (100 MHz, DMSO-d6); Ethyl 5-(2-chlorophenyl)-3,7-dimethyl-5H-thiazolo[3,2-a]pyrimidine-6-carboxylate (6d). Fig. S9. 1H NMR (400 MHz, DMSO-d6); Ethyl 5-(4-chlorophenyl)-3,7-dimethyl-5H-thiazolo[3,2-a]pyrimidine-6-carboxylate (6e). Fig. S10. 13C NMR (100 MHz, DMSO-d6); Ethyl 5-(4-chlorophenyl)-3,7-dimethyl-5H-thiazolo[3,2-a]pyrimidine-6-carboxylate (6e). Fig. S11. 1H NMR (400 MHz, DMSO-d6); Ethyl 5-(3-bromophenyl)-3,7-dimethyl-5H-thiazolo[3,2-a]pyrimidine-6-carboxylate (6f). Fig. S12. 13C NMR (100 MHz, DMSO-d6); Ethyl 5-(3-bromophenyl)-3,7-dimethyl-5H-thiazolo[3,2-a]pyrimidine-6-carboxylate (6f). Fig. S13. 1H NMR (400 MHz, DMSO-d6); Ethyl 3,7-dimethyl-5-(3-nitrophenyl)-5H-thiazolo[3,2-a]pyrimidine-6-carboxylate (6g). Fig. S14. 13C NMR (100 MHz, DMSO-d6); Ethyl 3,7-dimethyl-5-(3-nitrophenyl)-5H-thiazolo[3,2-a]pyrimidine-6-carboxylate (6g). Fig. S15. 1H NMR (400 MHz, DMSO-d6); Ethyl 3,7-dimethyl-5-(p-tolyl)-5H-thiazolo[3,2-a]pyrimidine-6-carboxylate (6h). Fig. S16. 13C NMR (100 MHz, DMSO-d6); Ethyl 3,7-dimethyl-5-(p-tolyl)-5H-thiazolo[3,2-a]pyrimidine-6-carboxylate (6h). Fig. S17. 1H NMR (400 MHz, DMSO-d6); [file 13065_2023_1077_MOESM1_ESM.docx]

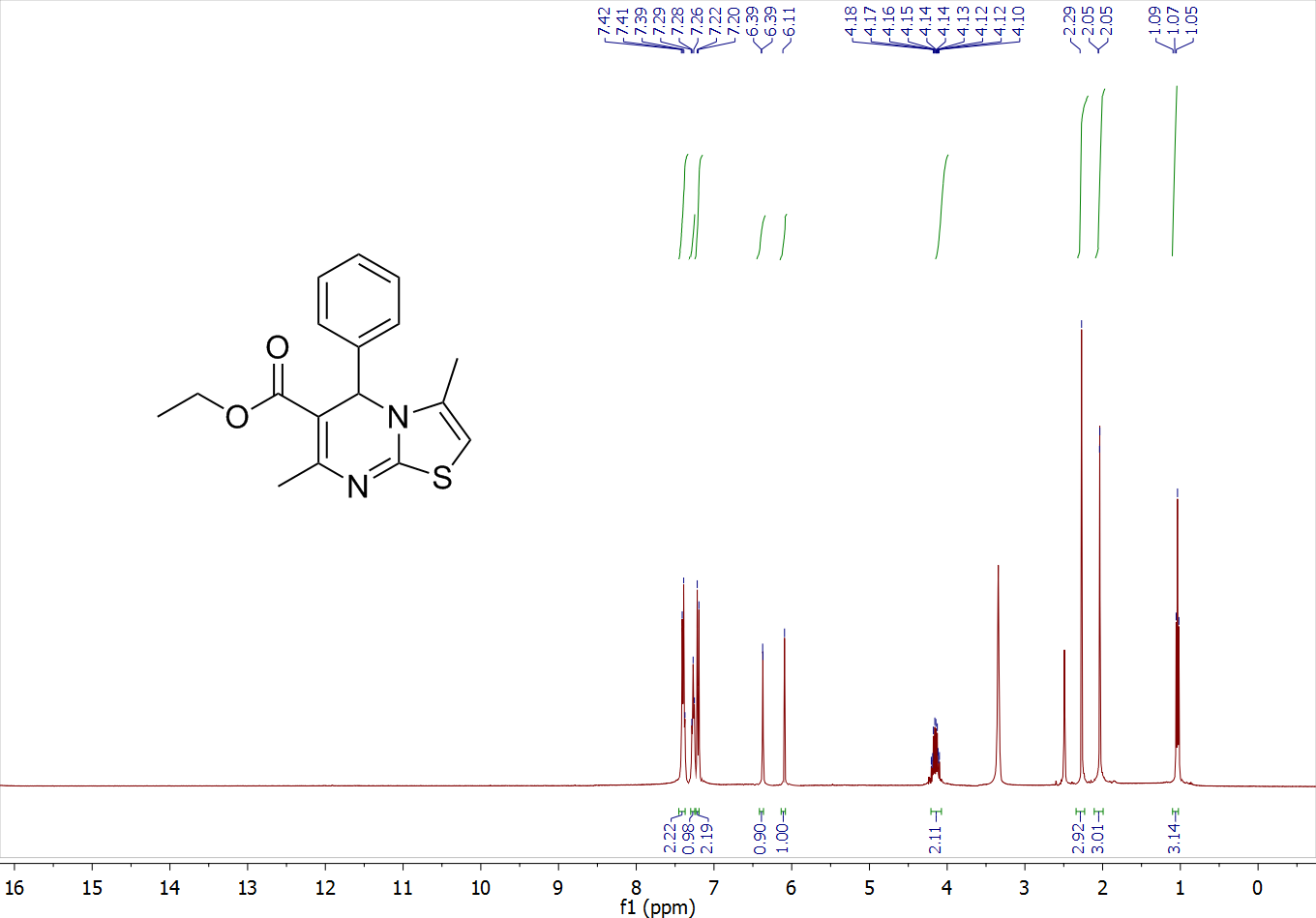


Fig. S1. ^1^H NMR (400 MHz, DMSO-*d_6_*); Ethyl 3,7-dimethyl-5-phenyl-5H-thiazolo[3,2-a]pyrimidine-6-carboxylate (**6a**).


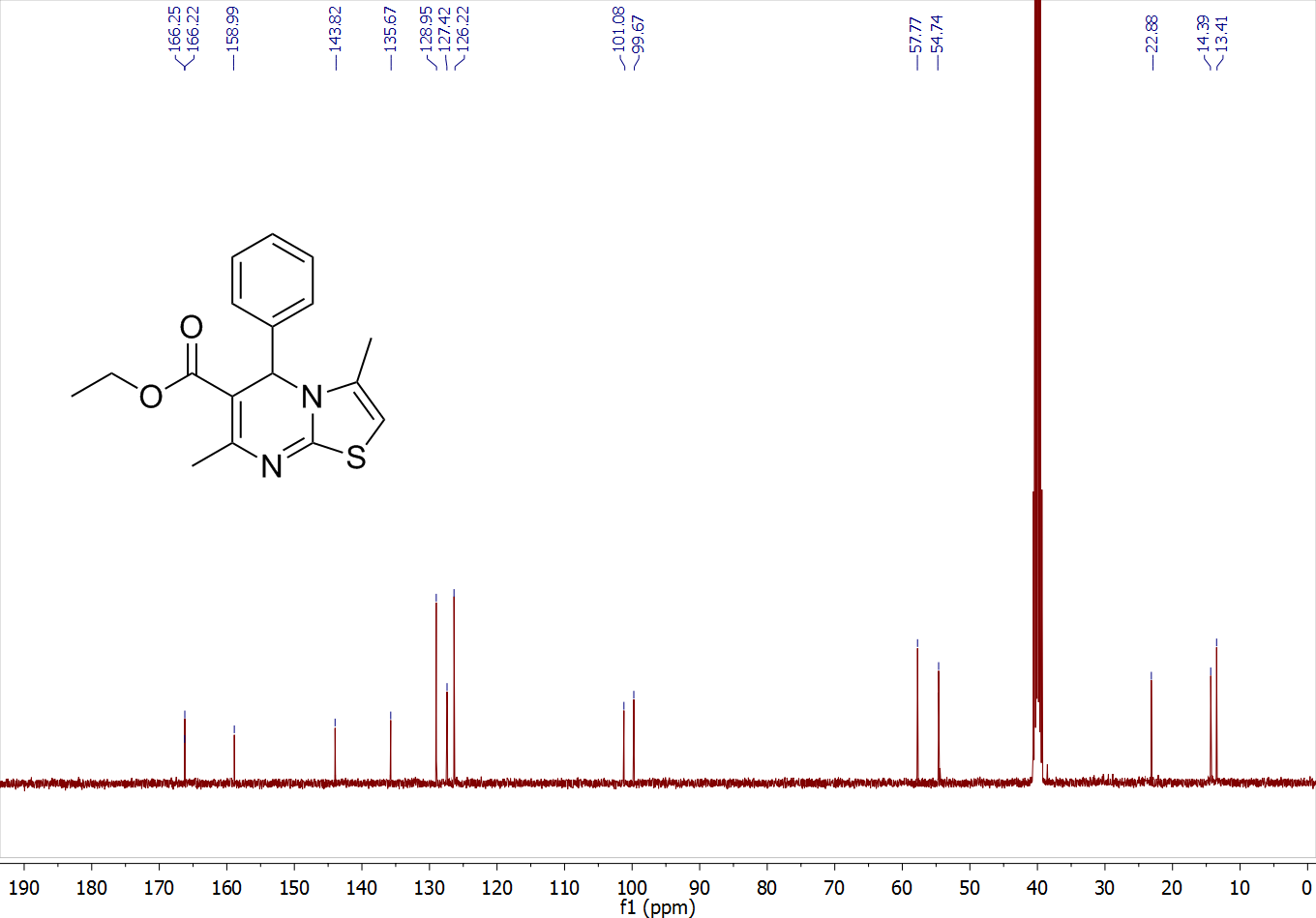


Fig. S2. ^13^C NMR (100 MHz, DMSO-d_6_); Ethyl 3,7-dimethyl-5-phenyl-5H-thiazolo[3,2-a]pyrimidine-6-carboxylate (**6a**)


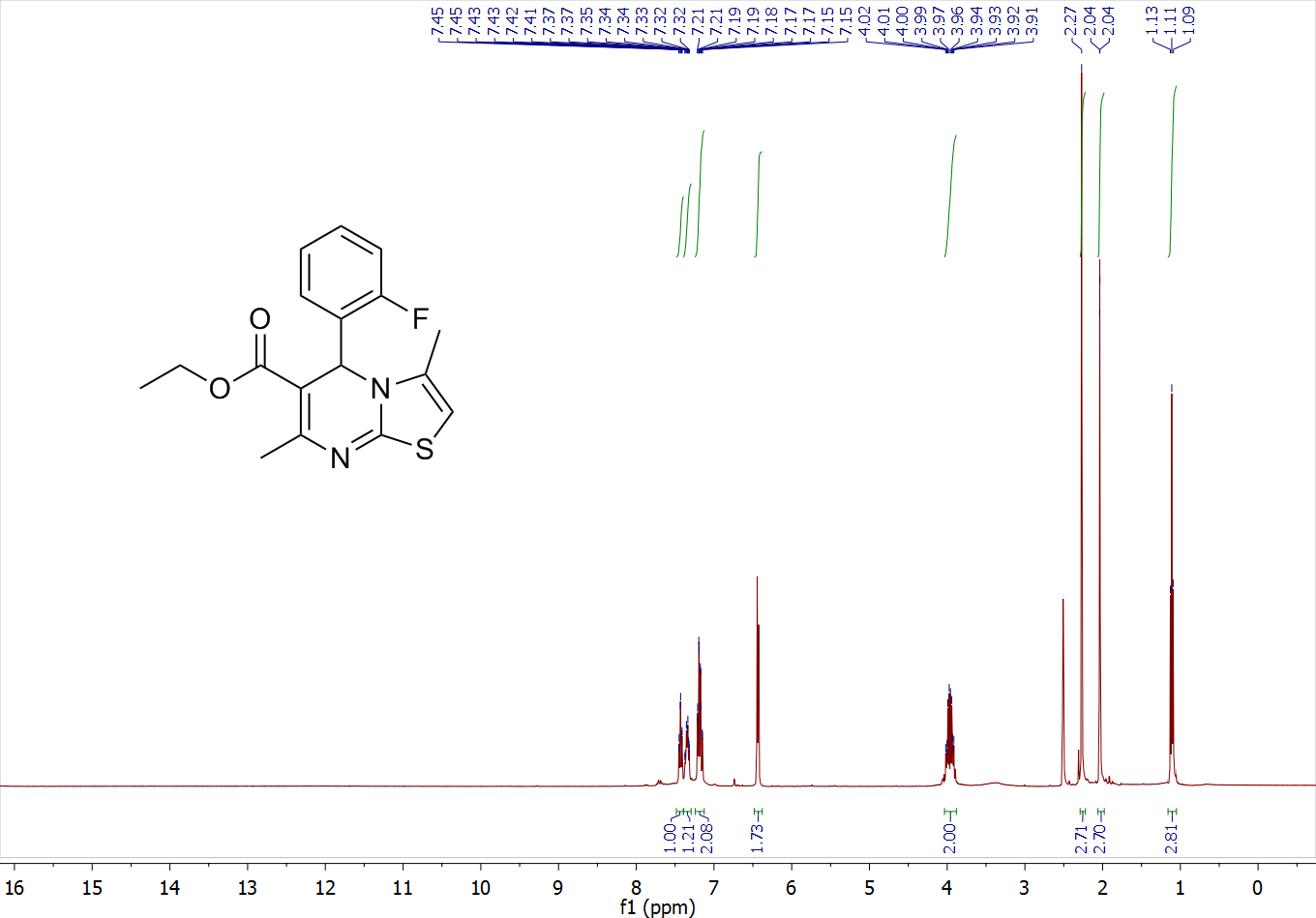


Fig. S3. ^1^H NMR (400 MHz, DMSO-d_6_); Ethyl 5-(2-fluorophenyl)-3,7-dimethyl-5H-thiazolo[3,2-a]pyrimidine-6-carboxylate (**6b**).


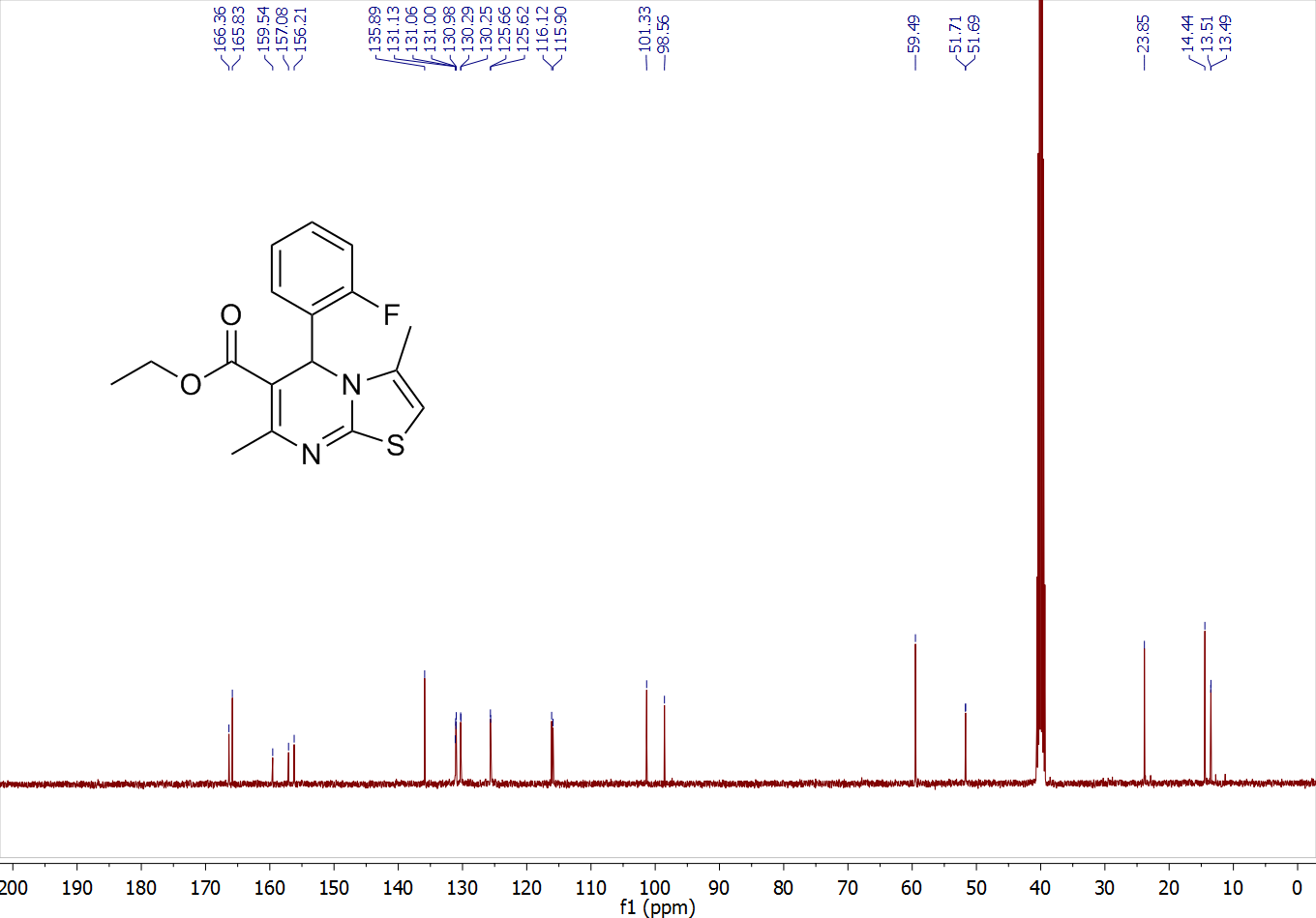


Fig. S4. ^13^C NMR (100 MHz, DMSO-d_6_); Ethyl 5-(2-fluorophenyl)-3,7-dimethyl-5H-thiazolo[3,2-a]pyrimidine-6-carboxylate (**6b**).


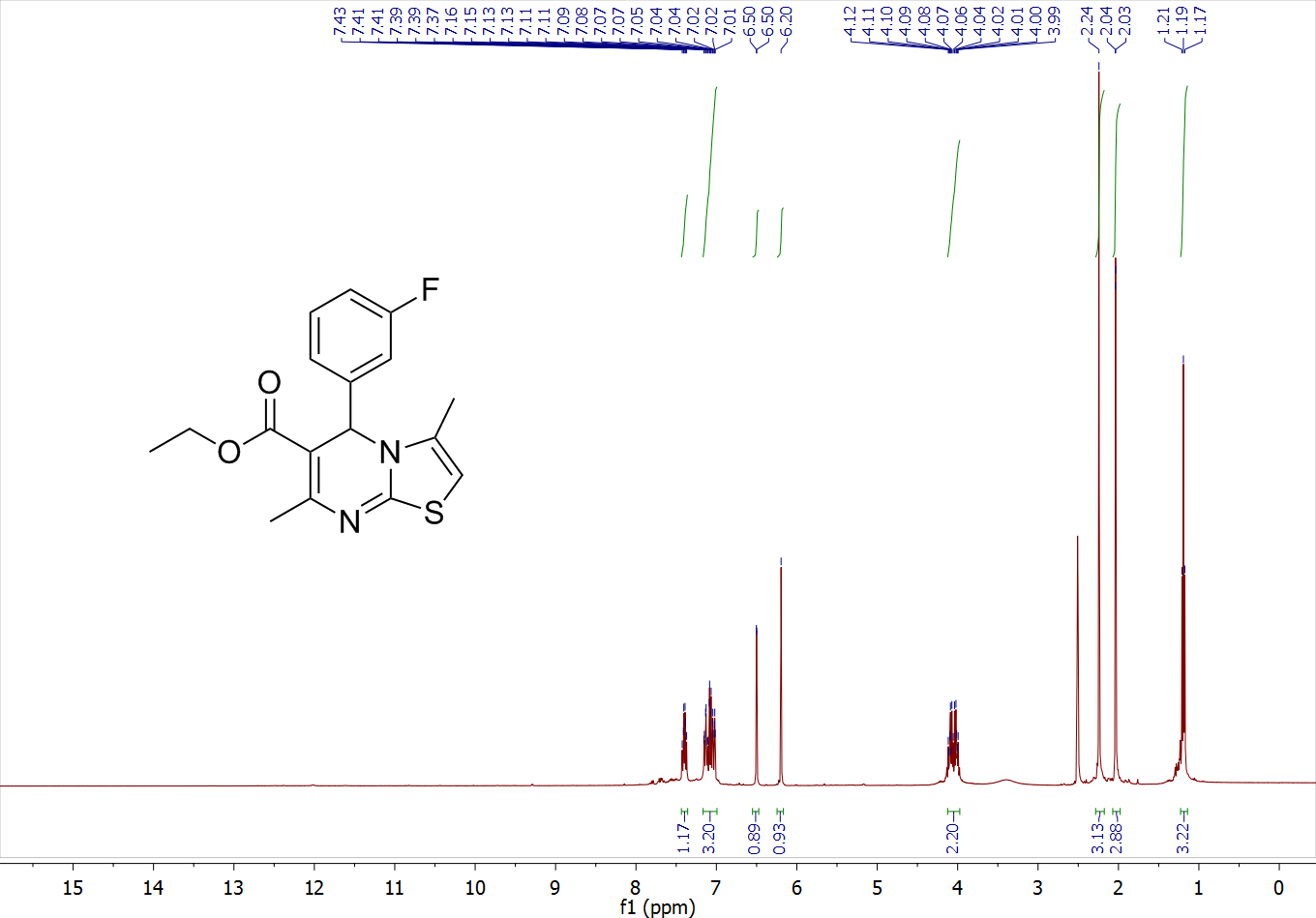


Fig. S5. ^1^H NMR (400 MHz, DMSO-d_6_); Ethyl 5-(3-fluorophenyl)-3,7-dimethyl-5H-thiazolo[3,2-a]pyrimidine-6-carboxylate (**6c**)


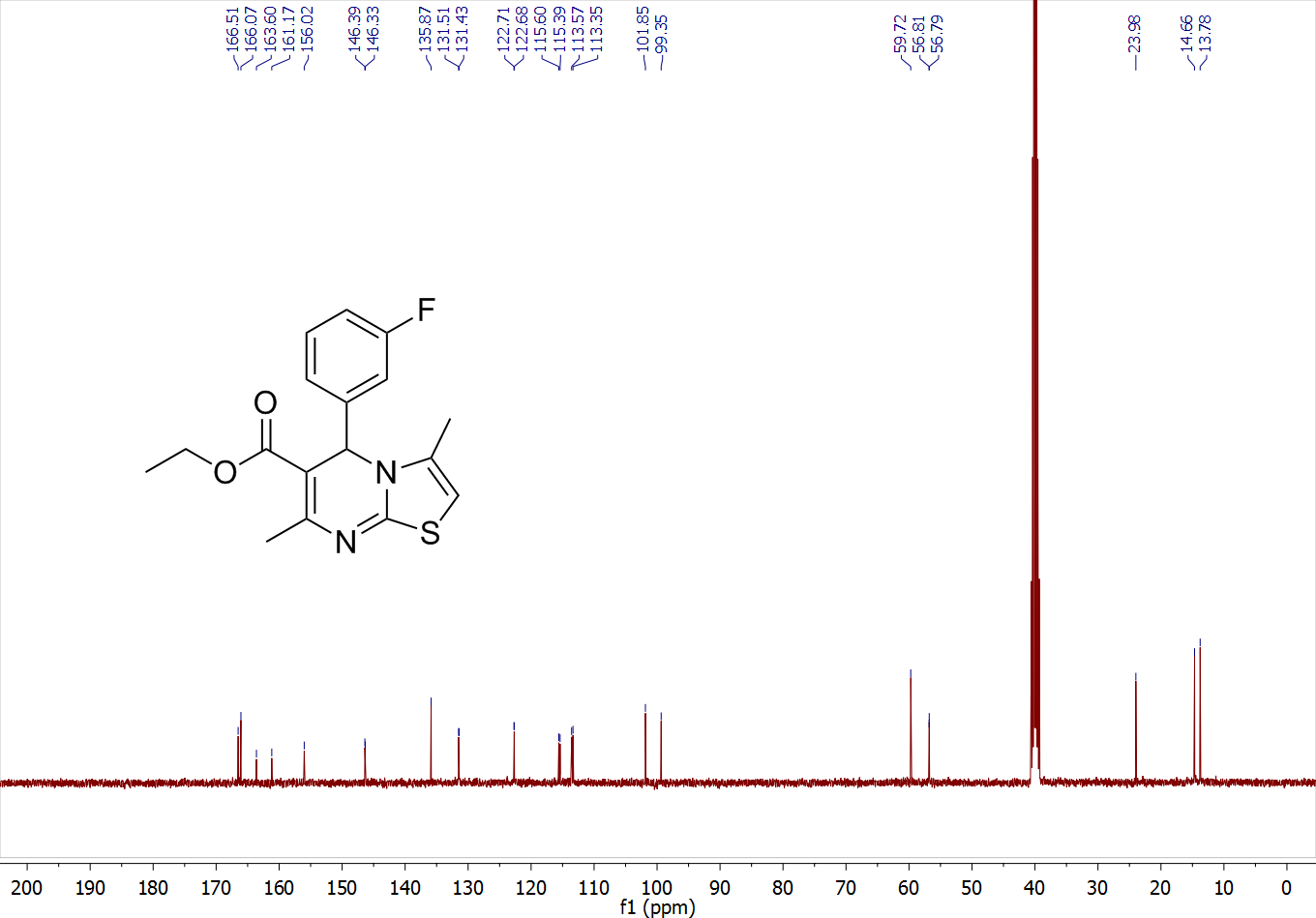


Fig. S6. ^13^C NMR (100 MHz, DMSO-d_6_); Ethyl 5-(3-fluorophenyl)-3,7-dimethyl-5H-thiazolo[3,2-a]pyrimidine-6-carboxylate (**6c**)


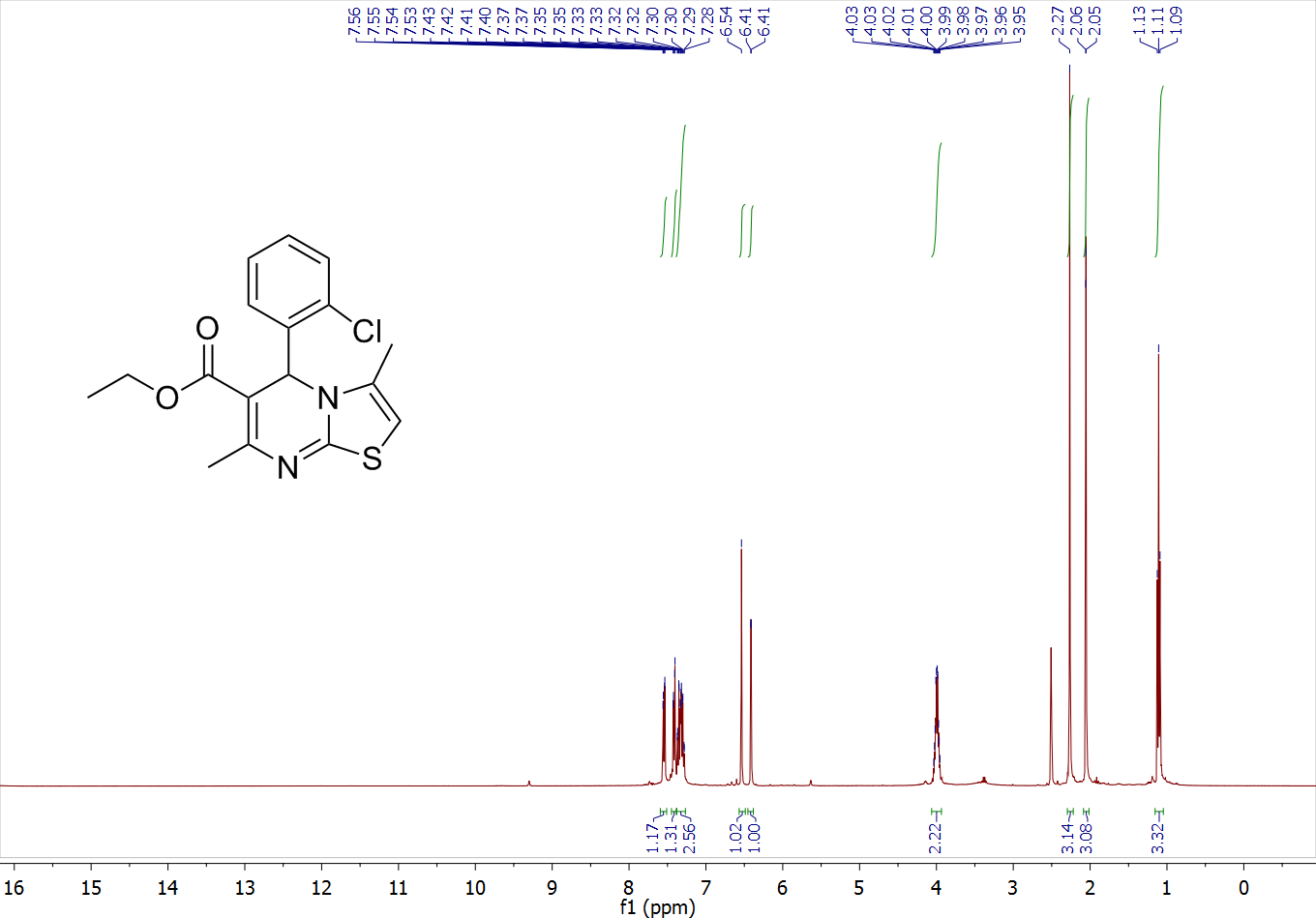


Fig. S7. ^1^H NMR (400 MHz, DMSO-*d_6_)*; Ethyl 5-(2-chlorophenyl)-3,7-dimethyl-5*H*-thiazolo[3,2-*a*]pyrimidine-6-carboxylate (**6d**)


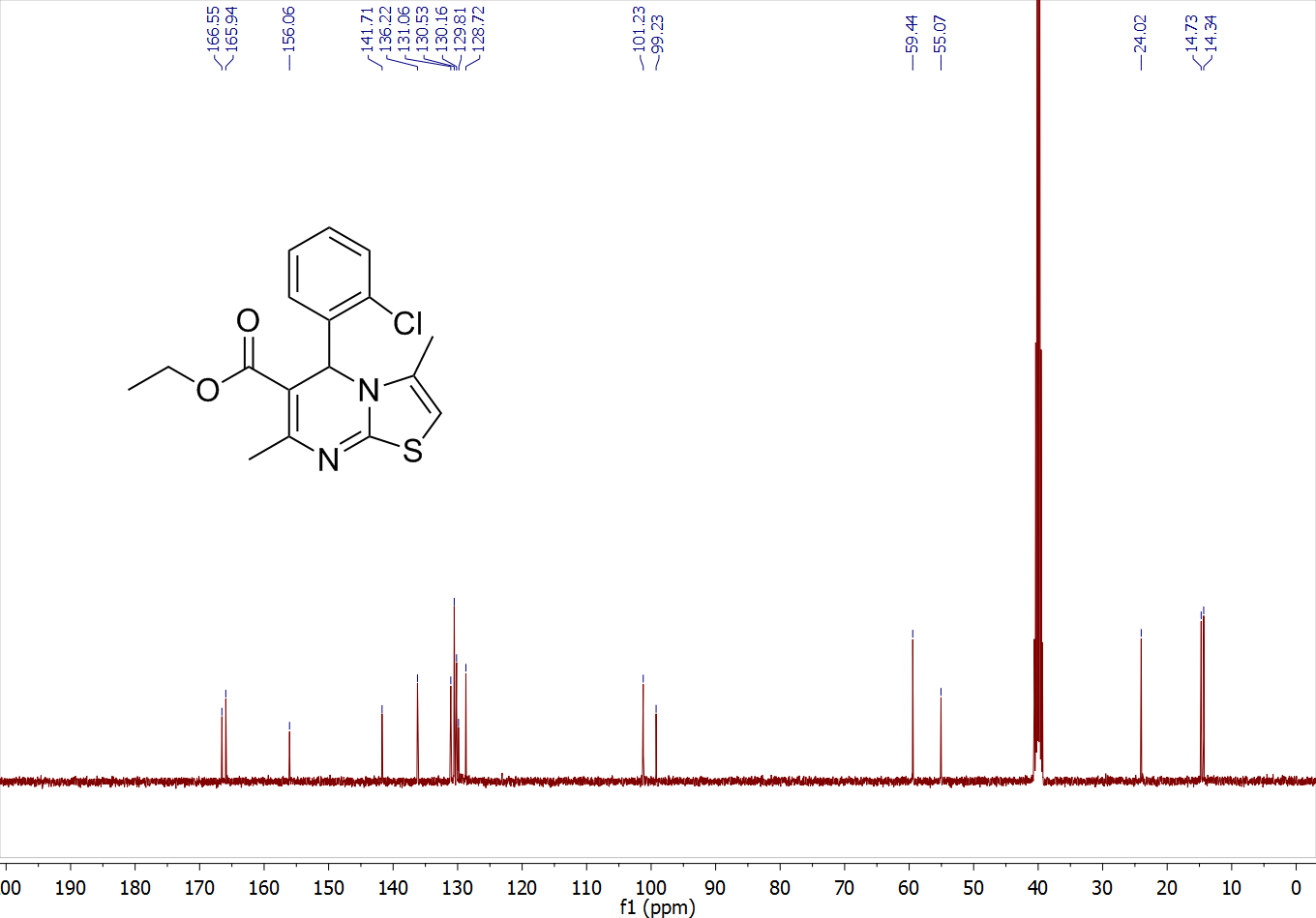


Fig. S8*.* ^13^C NMR (100 MHz, DMSO-*d_6_);* Ethyl 5-(2-chlorophenyl)-3,7-dimethyl-5*H*-thiazolo[3,2-*a*]pyrimidine-6-carboxylate (**6d**)

**
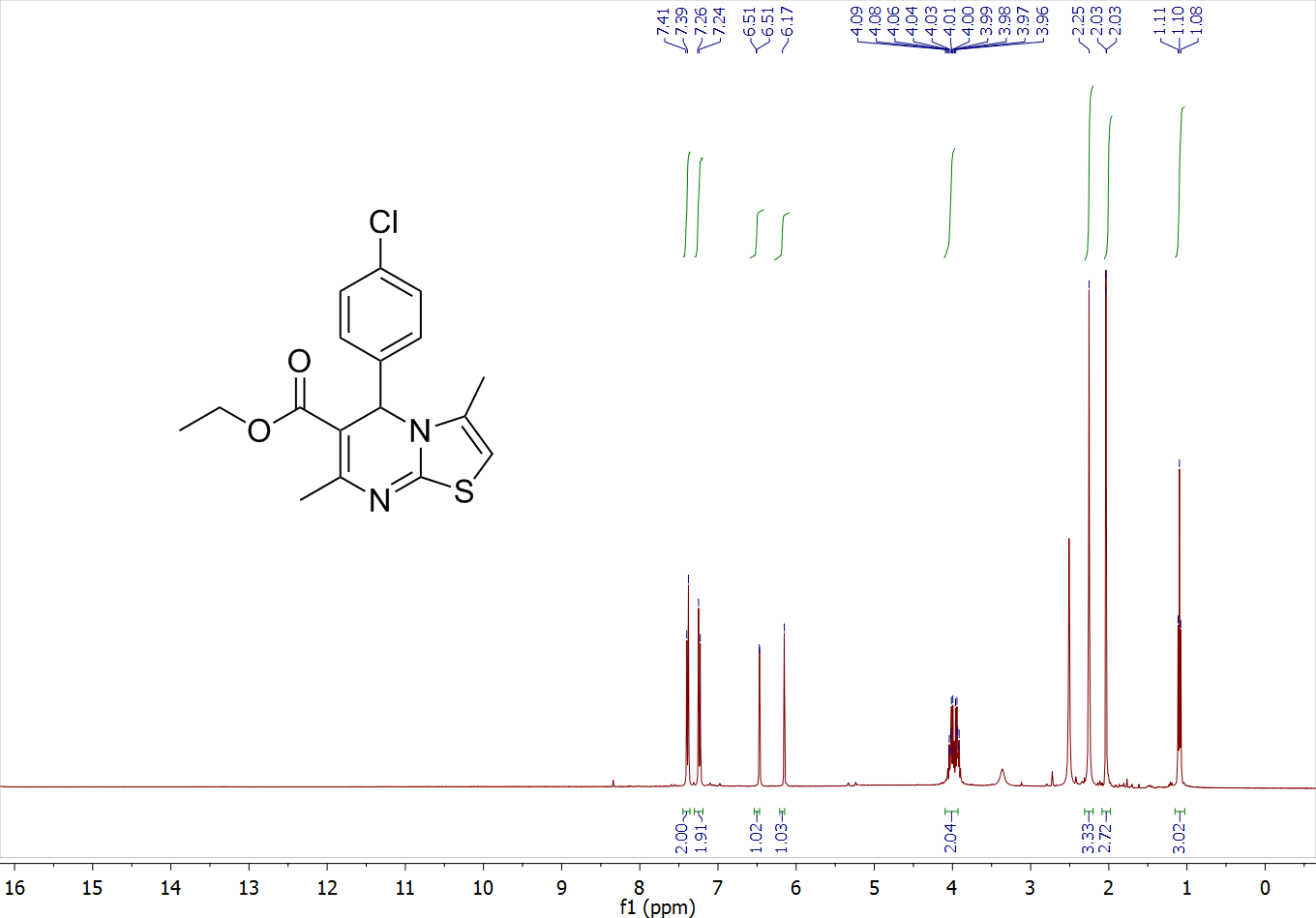
**

Fig. S9. ^1^H NMR (400 MHz, DMSO-*d_6_*); Ethyl 5-(4-chlorophenyl)-3,7-dimethyl-5H-thiazolo[3,2-a]pyrimidine-6-carboxylate (**6e**)

**
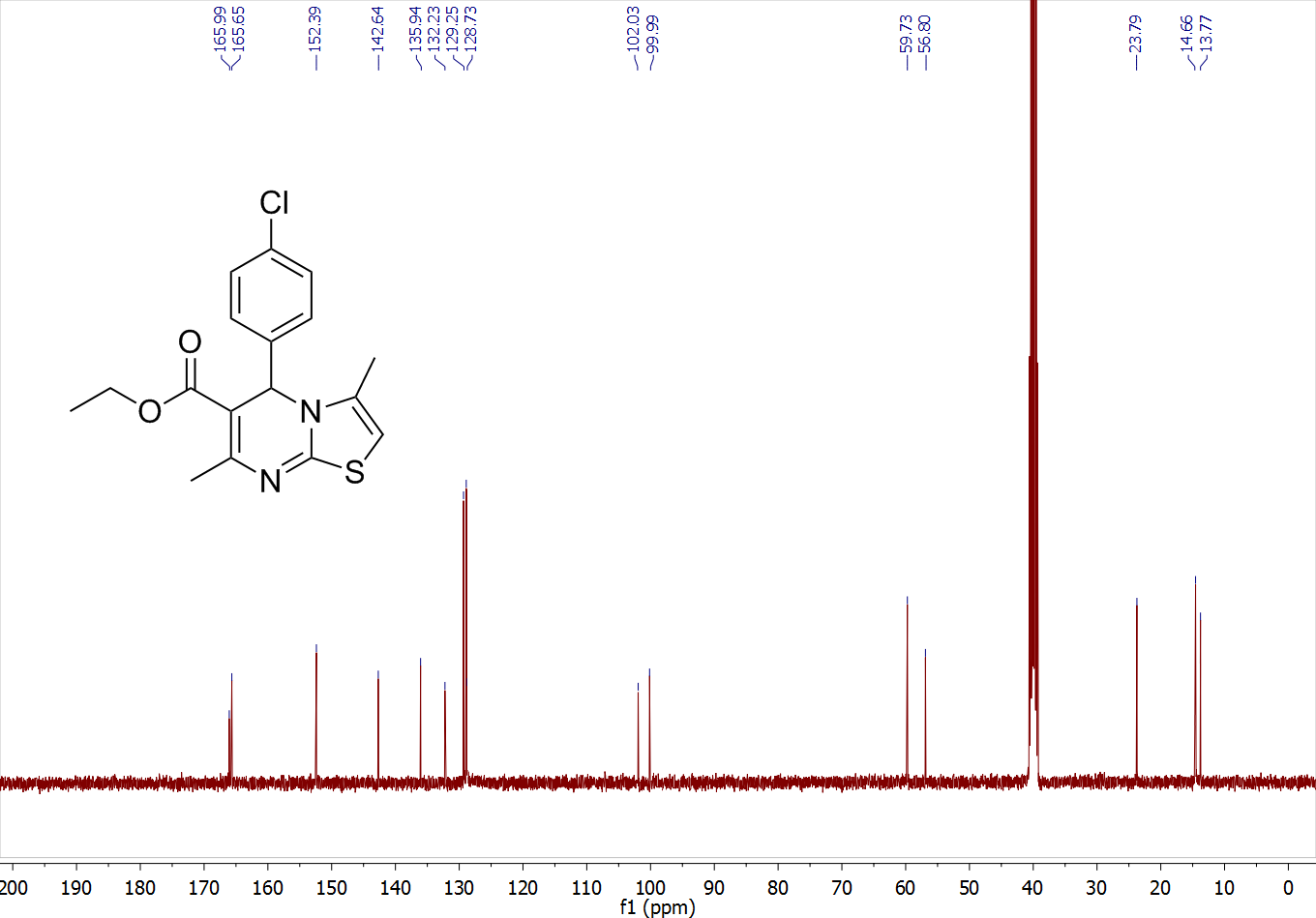
**

Fig. S10*.* ^13^C NMR (100 MHz, DMSO-*d_6_*)*;* Ethyl 5-(4-chlorophenyl)-3,7-dimethyl-5H-thiazolo[3,2-a]pyrimidine-6-carboxylate (**6e**)


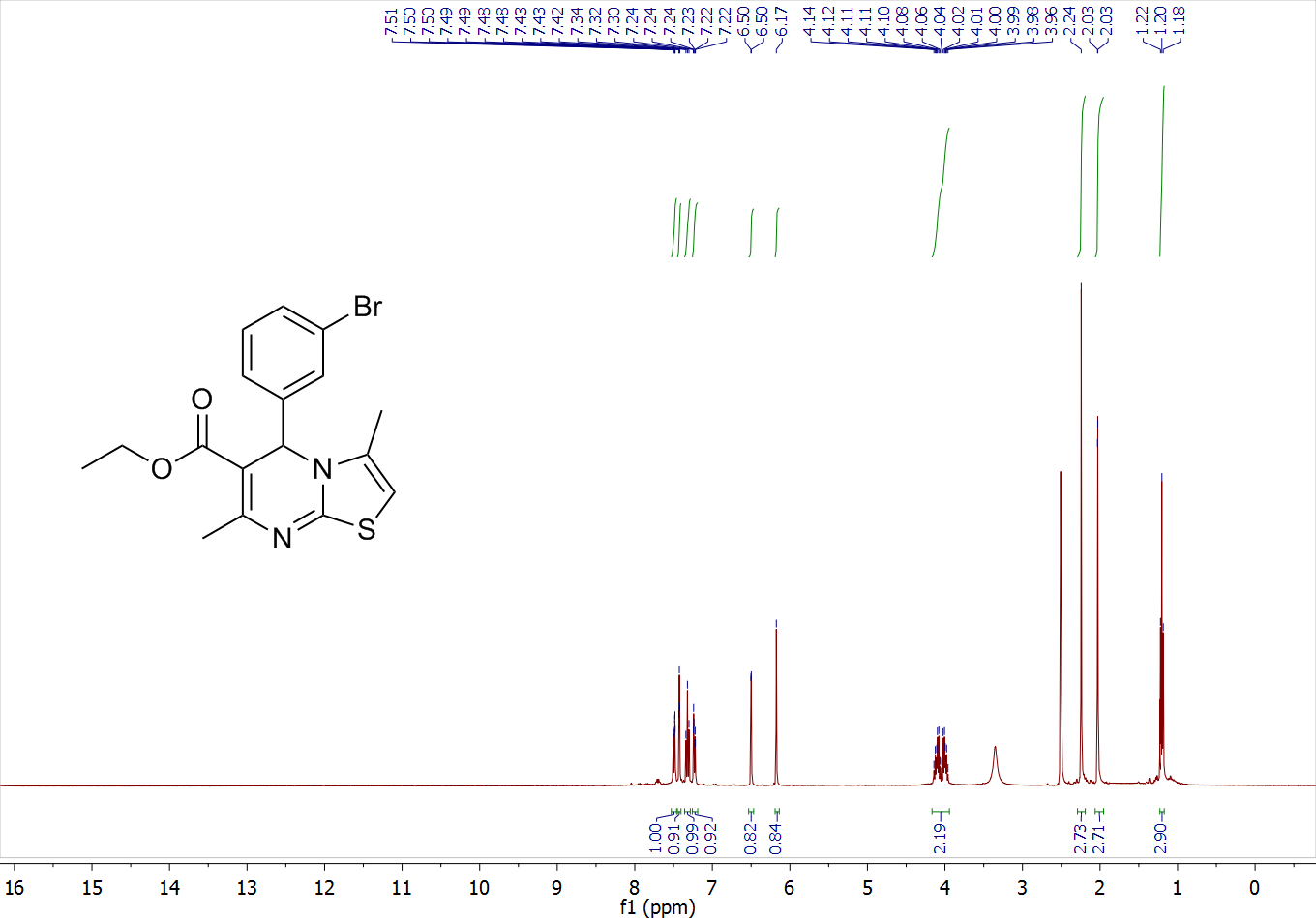


Fig. S11. ^1^H NMR (400 MHz, DMSO-*d_6_*); Ethyl 5-(3-bromophenyl)-3,7-dimethyl-5H-thiazolo[3,2-a]pyrimidine-6-carboxylate (**6f**)


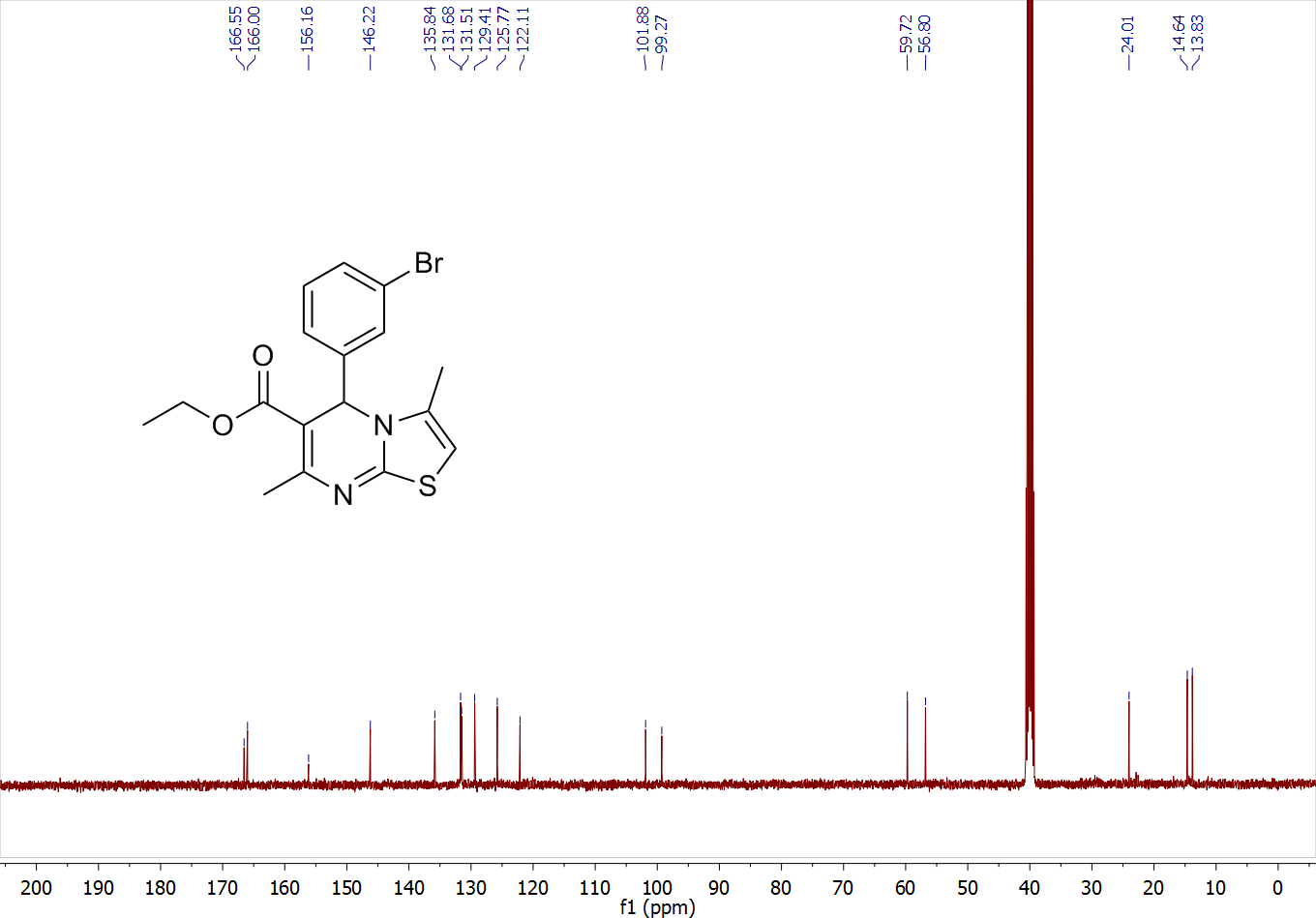


Fig. S12*.* ^13^C NMR (100 MHz, DMSO-*d_6_);* Ethyl 5-(3-bromophenyl)-3,7-dimethyl-5H-thiazolo[3,2-a]pyrimidine-6-carboxylate (**6f**)


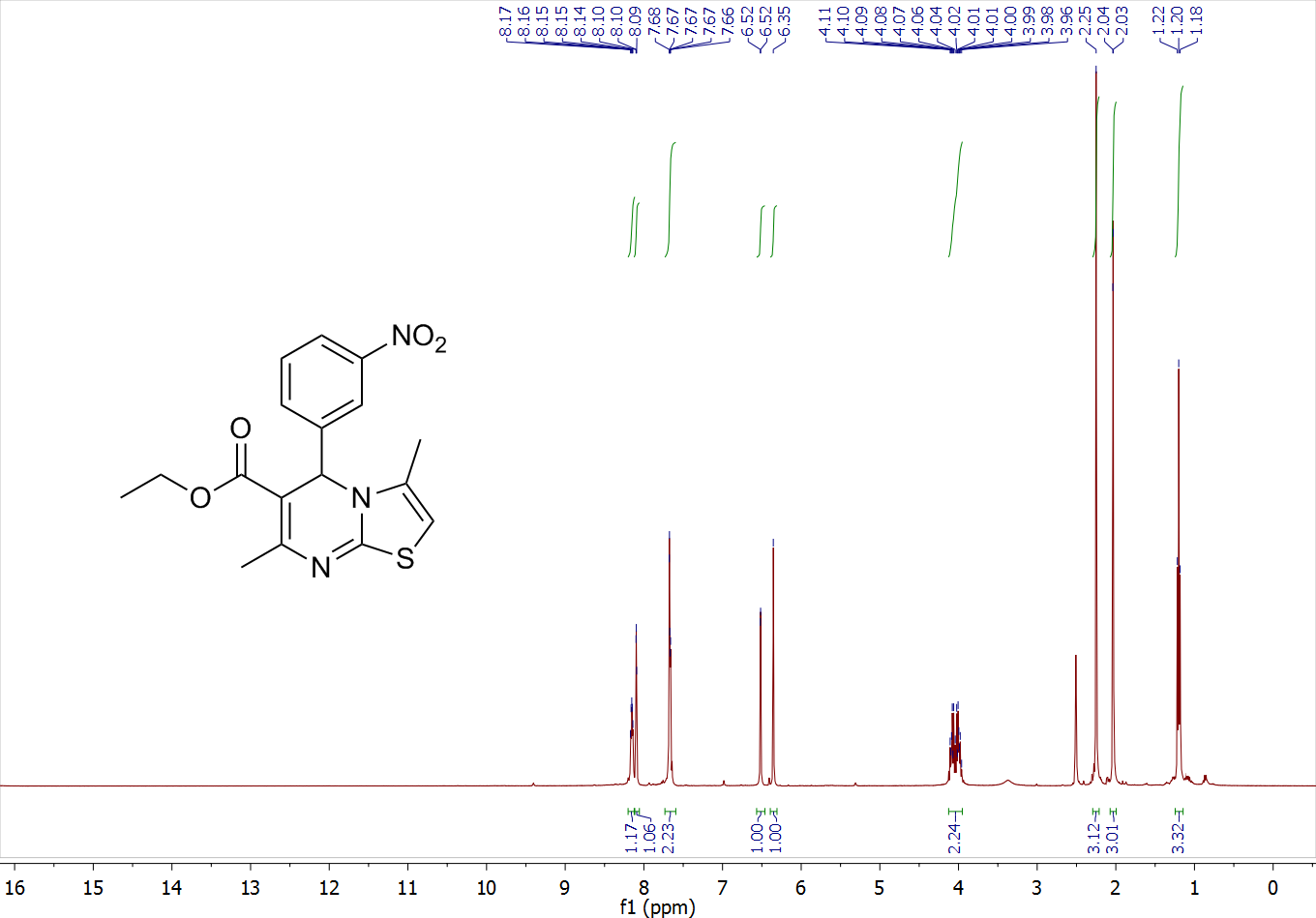


Fig. S13. ^1^H NMR (400 MHz, DMSO-*d_6_*); Ethyl 3,7-dimethyl-5-(3-nitrophenyl)-5H-thiazolo[3,2-a]pyrimidine-6-carboxylate (**6g**)


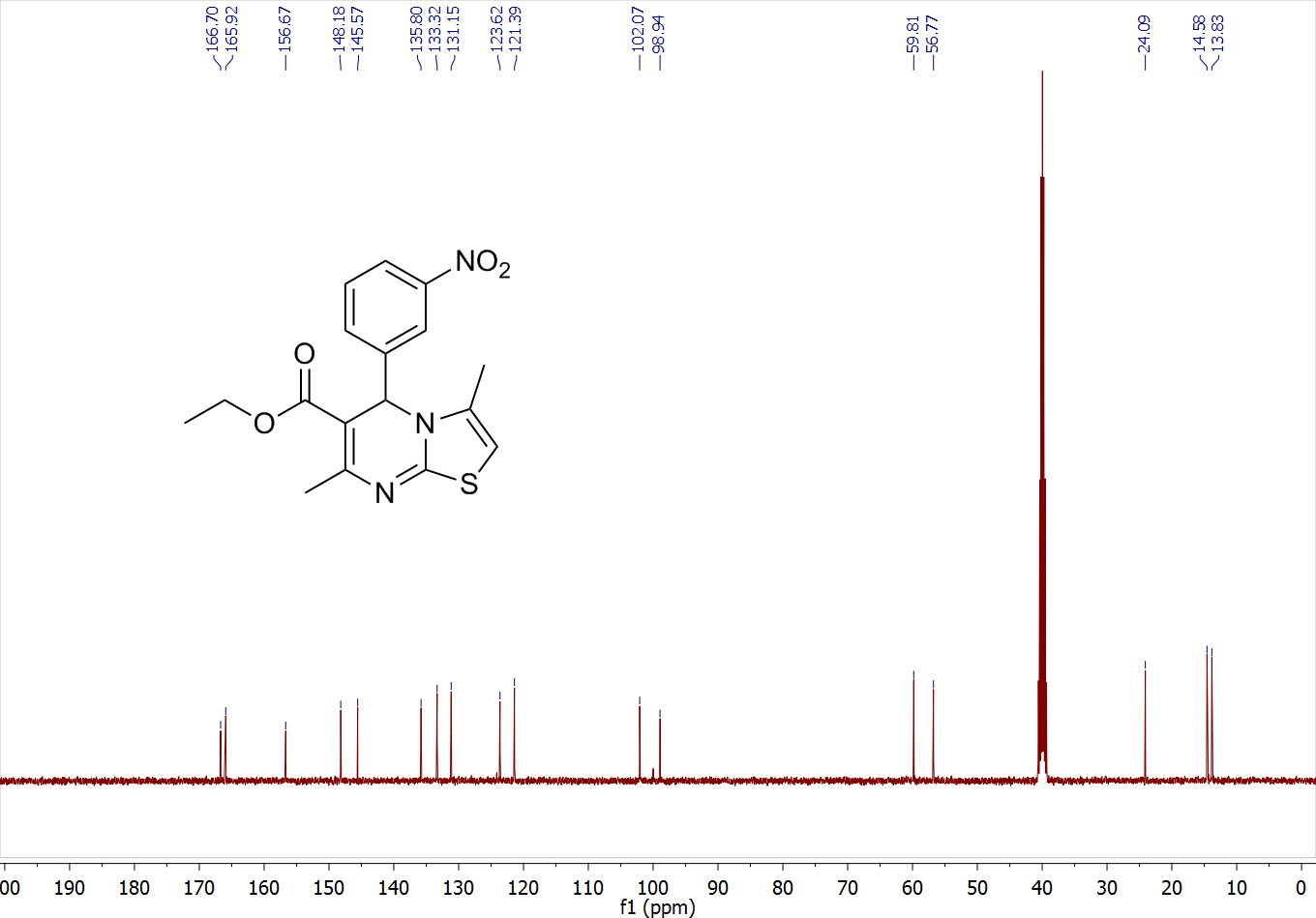


Fig. S14*.* ^13^C NMR (100 MHz, DMSO-*d_6_);* Ethyl 3,7-dimethyl-5-(3-nitrophenyl)-5*H*-thiazolo[3,2-*a*]pyrimidine-6-carboxylate (**6g**)


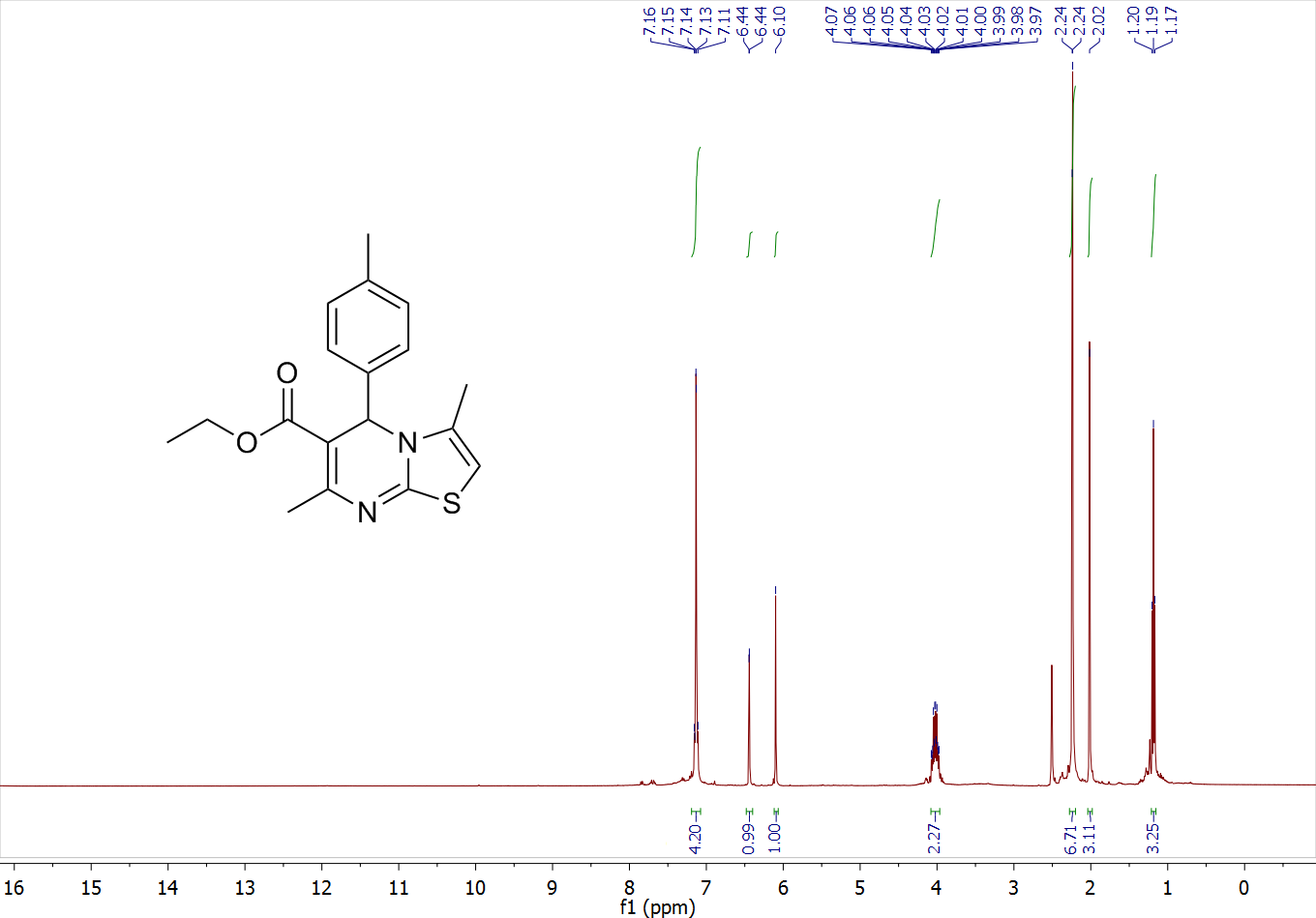


Fig. S15. ^1^H NMR (400 MHz, DMSO-*d_6_*); Ethyl 3,7-dimethyl-5-(p-tolyl)-5H-thiazolo[3,2-a]pyrimidine-6-carboxylate (**6h**)


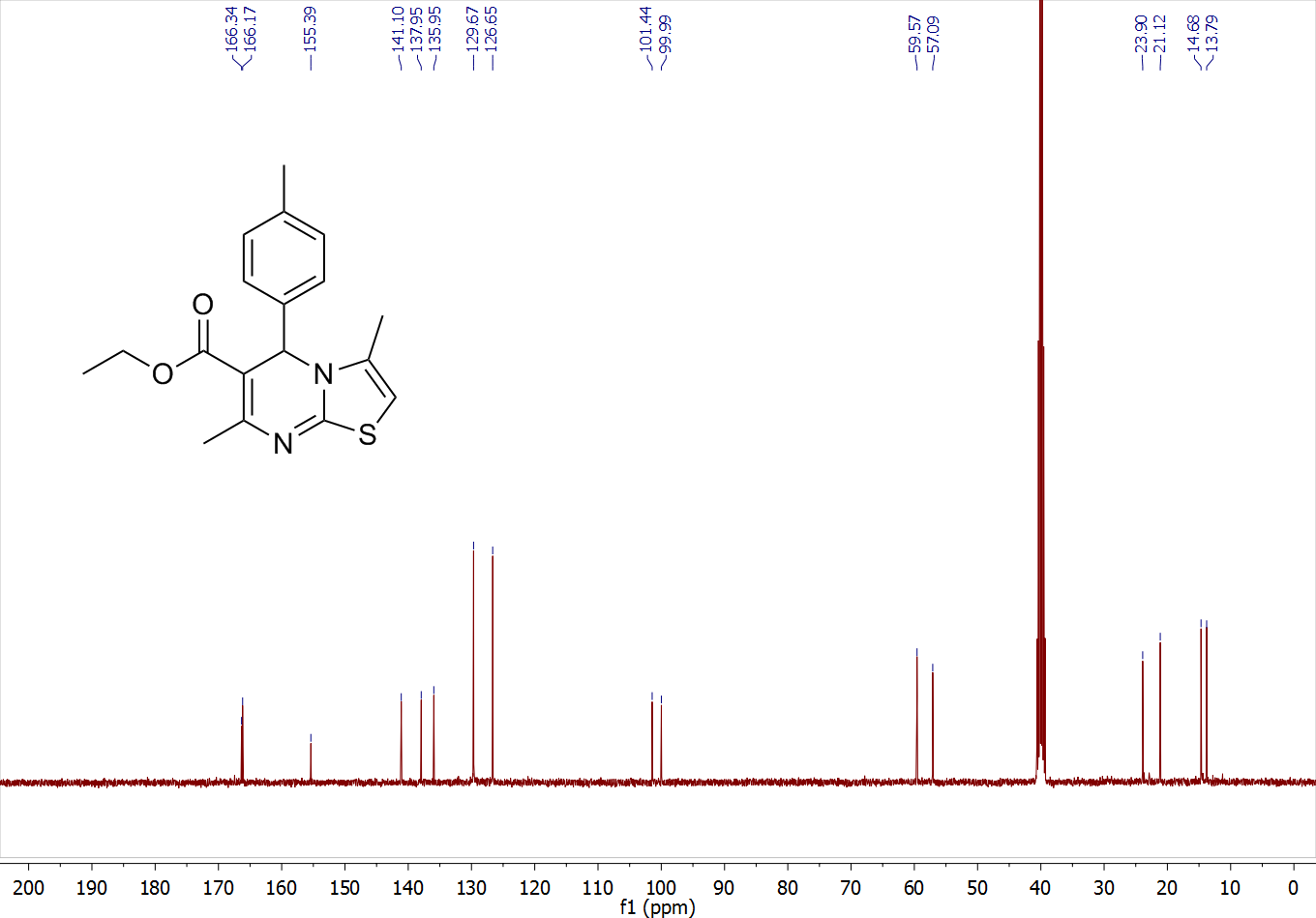


Fig. S16. ^13^C NMR (100 MHz, DMSO-*d_6_*); Ethyl 3,7-dimethyl-5-(p-tolyl)-5H-thiazolo[3,2-a]pyrimidine-6-carboxylate (**6h**)


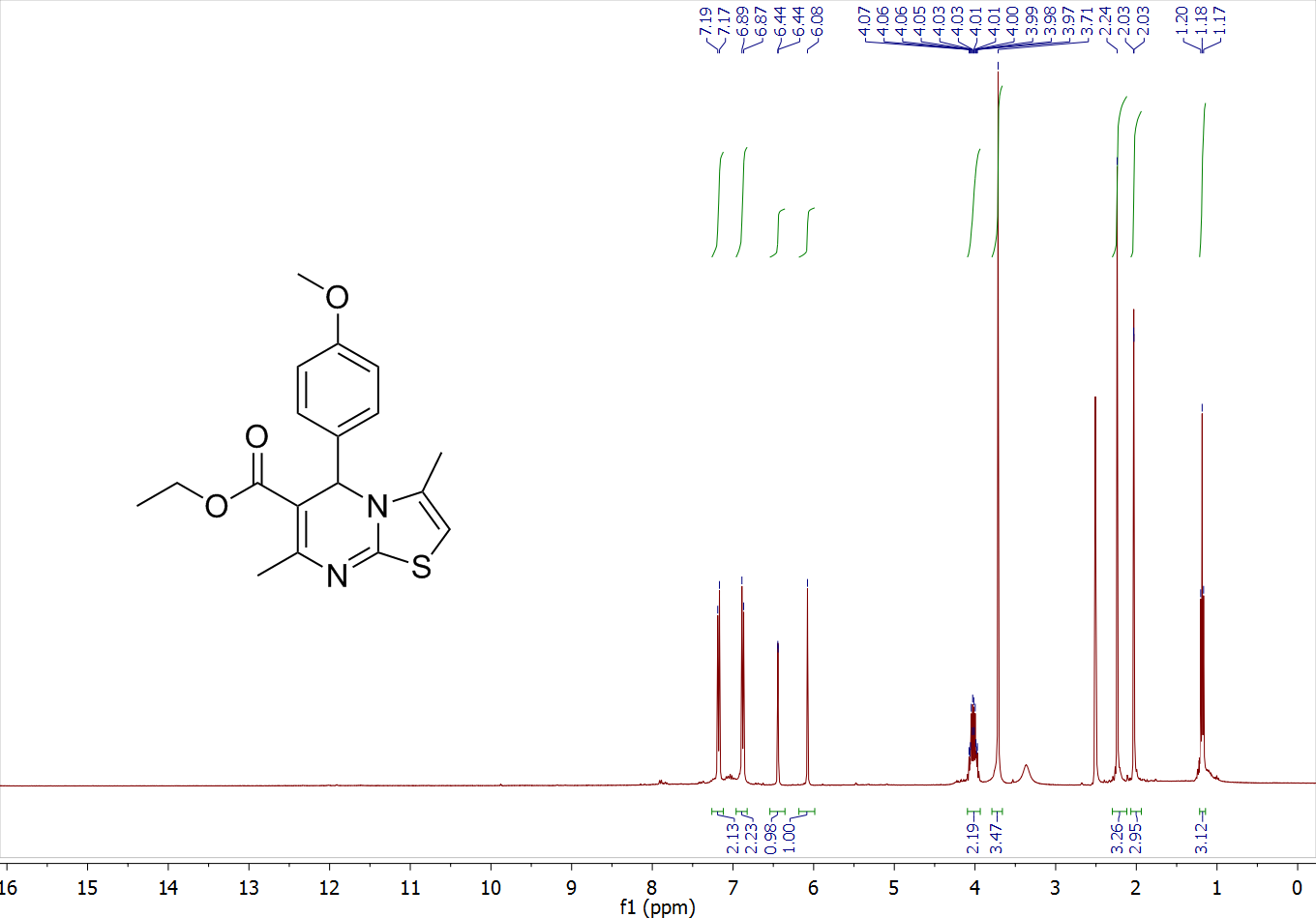


Fig. S17. ^1^H NMR (400 MHz, DMSO-*d_6_*); Ethyl 5-(4-methoxyphenyl)-3,7-dimethyl-5H-thiazolo[3,2-a]pyrimidine-6-carboxylate (**6i**)


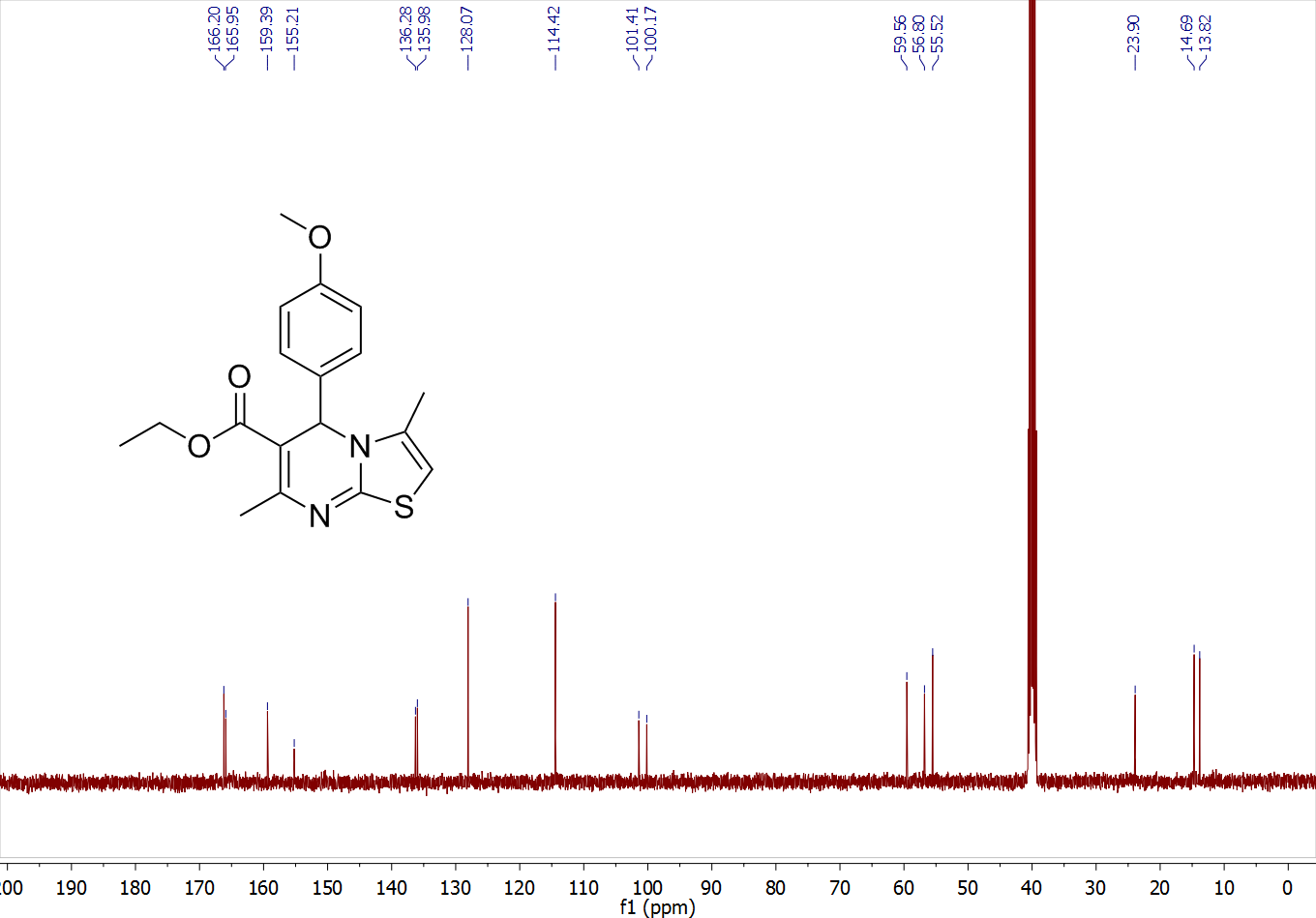


Fig. S18. ^13^C NMR (100 MHz, DMSO-*d_6_*); Ethyl 5-(4-methoxyphenyl)-3,7-dimethyl-5H-thiazolo[3,2-a]pyrimidine-6-carboxylate (**6i**)


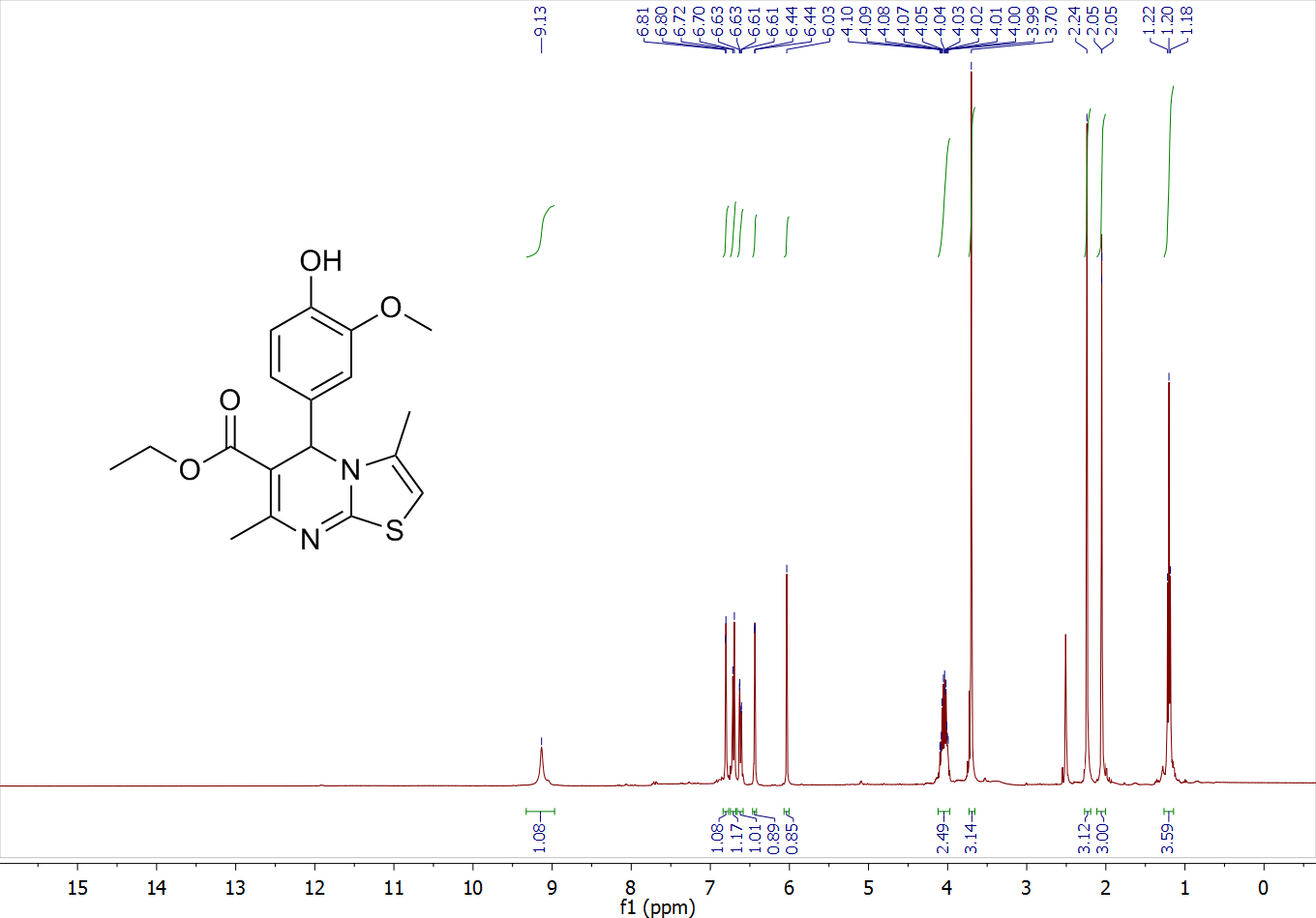


Fig. S19. ^1^H NMR (400 MHz, DMSO-*d_6_*); Ethyl 5-(4-hydroxy-3-methoxyphenyl)-3,7-dimethyl-5H-thiazolo[3,2-a]pyrimidine-6-carboxylate (**6j**)


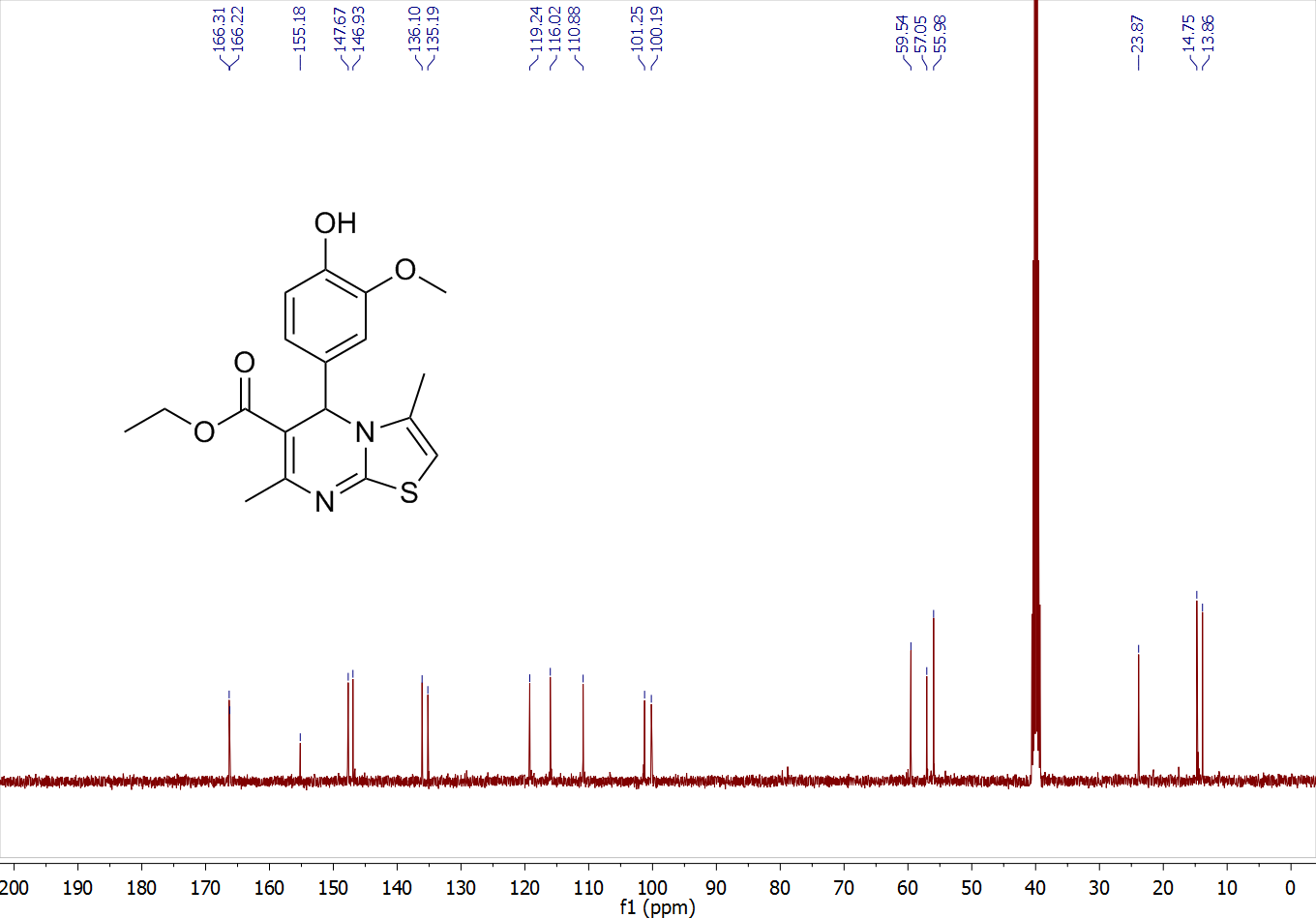


Fig. S20. ^13^C NMR (100 MHz, DMSO-*d_6_*); Ethyl 5-(4-hydroxy-3-methoxyphenyl)-3,7-dimethyl-5H-thiazolo[3,2-a]pyrimidine-6-carboxylate (**6j**).

Table S1. Results of molecular docking study of **6a-j** against tyrosinase binding site

| **Entry** | **Binding energy** | **Moiety of compound** | **Residue** | **Type of interactions** |
| --- | --- | --- | --- | --- |
| **6b** | -5.460 | C=O of ethyl acetate  O-Et of ethyl acetate | Lys180  Lys180 | H-bound  Salt bridge |
| **6c** | -5.117  -4.675 | C=O of ethyl acetate  O-Et of ethyl acetate | Lys180  Lys180 | H-bound  Salt bridge |
| **6d** | -5.279 | C=O of ethyl acetate  O-Et of ethyl acetate | Lys180  Lys180 | H-bound  Salt bridge |
| **6e** | -5.790 | C=O of ethyl acetate  O-Et of ethyl acetate | Lys180  Lys180 | H-bound  Salt bridge |
| **6f** | -4.497 | C=O of ethyl acetate | Lys180 | H-bound |
| **6g** | -4.586 | C=O of ethyl acetate  O-Et of ethyl acetate | Lys180  Lys180 | H-bound  Salt bridge |
| **6h** | -4.672 | C=O of ethyl acetate | Lys180 | H-bound |
| **6i** | -5.519 | C=O of ethyl acetate  O-Et of ethyl acetate | Lys180  Lys180 | H-bound  Salt bridge |
| **6j** | -5.452 | Phenyl  O-Et of ethyl acetate  OH | Lys180  Lys180  Thr197 | Pi-pi stacking  Salt bridge  H-bound |
| **Tropolone** | -6.064  -4.898 | C=O  C=O | His178  Lys180 | H-bound  H-bound |
